# Methylome and transcriptome data integration reveals potential roles of DNA methylation and candidate biomarkers of cow *Streptococcus uberis* subclinical mastitis

**DOI:** 10.1186/s40104-022-00779-z

**Published:** 2022-11-07

**Authors:** Mengqi Wang, Nathalie Bissonnette, Mario Laterrière, Pier-Luc Dudemaine, David Gagné, Jean-Philippe Roy, Xin Zhao, Marc-André Sirard, Eveline M. Ibeagha-Awemu

**Affiliations:** 1 Sherbrooke Research and Development Centre, Agriculture and Agri-Food Canada, Sherbrooke, Quebec Canada; 2 Department of Animal Science, Laval University, Quebec, Quebec Canada; 3 Quebec Research and Development Centre, Agriculture and Agri-Food Canada, Quebec, Quebec Canada; 4 Department of Clinical Sciences, Université de Montréal, St-Hyacinthe, Québec Canada; 5 Department of Animal Science, McGill University, Ste-Anne-De-Bellevue, Quebec Canada

**Keywords:** Discriminant biomarkers, Gene expression, Genome-wide DNA methylation pattern, Immune processes and pathways, Methylation haplotype block, Milk somatic cell, *Streptococcus uberis*, Subclinical mastitis

## Abstract

**Background:**

Mastitis caused by different pathogens including *Streptococcus uberis* (*S. uberis*) is responsible for huge economic losses to the dairy industry. In order to investigate the potential genetic and epigenetic regulatory mechanisms of subclinical mastitis due to *S. uberis*, the DNA methylome (whole genome DNA methylation sequencing) and transcriptome (RNA sequencing) of milk somatic cells from cows with naturally occurring *S. uberis* subclinical mastitis and healthy control cows (*n* = 3/group) were studied.

**Results:**

Globally, the DNA methylation levels of CpG sites were low in the promoters and first exons but high in inner exons and introns. The DNA methylation levels at the promoter, first exon and first intron regions were negatively correlated with the expression level of genes at a whole-genome-wide scale. In general, DNA methylation level was lower in *S. uberis*-positive group (SUG) than in the control group (CTG). A total of 174,342 differentially methylated cytosines (DMCs) (FDR < 0.05) were identified between SUG and CTG, including 132,237, 7412 and 34,693 DMCs in the context of CpG, CHG and CHH (H = A or T or C), respectively. Besides, 101,612 methylation haplotype blocks (MHBs) were identified, including 451 MHBs that were significantly different (dMHB) between the two groups. A total of 2130 differentially expressed (DE) genes (1378 with up-regulated and 752 with down-regulated expression) were found in SUG. Integration of methylome and transcriptome data with MethGET program revealed 1623 genes with significant changes in their methylation levels and/or gene expression changes (MetGDE genes, MethGET *P*-value < 0.001). Functional enrichment of genes harboring ≥ 15 DMCs, DE genes and MetGDE genes suggest significant involvement of DNA methylation changes in the regulation of the host immune response to *S. uberis* infection, especially cytokine activities. Furthermore, discriminant correlation analysis with DIABLO method identified 26 candidate biomarkers, including 6 DE genes, 15 CpG-DMCs and 5 dMHBs that discriminated between SUG and CTG.

**Conclusion:**

The integration of methylome and transcriptome of milk somatic cells suggests the possible involvement of DNA methylation changes in the regulation of the host immune response to subclinical mastitis due to *S. uberis*. The presented genetic and epigenetic biomarkers could contribute to the design of management strategies of subclinical mastitis and breeding for mastitis resistance.

**Supplementary Information:**

The online version contains supplementary material available at 10.1186/s40104-022-00779-z.

## Background

Bovine mastitis, defined as an inflammation of the mammary gland, is one of the most prevalent diseases in the dairy industry [1]. Mastitis is considered a serious problem because it not only causes huge economic losses (through reduced milk yield and quality, early culling and corresponding treatment costs), but also threatens public health [1, 2]. *Streptococcus uberis* (*S. uberis*) is a bacteria classified as a major environmental pathogen associated with subclinical and clinical mastitis in lactating cows, non-lactating cows and heifers [3, 4]. Because it is ubiquitous in the cow’s environment (e.g., present in water, feces, barn environment, bedding material, etc.), on different parts of the cow (skin, lips, teat canal, oral cavity, infected mammary gland and gut) and milking machine [5–7], the mammary glands are continually exposed to *S. uberis* during the lactating and non-lactating periods. Moreover, *S. uberis* originating from infected cows contribute to a fecal-oral transmission cycle that could help *S. uberis* to persist on pasture [8, 9].

The invasion of mastitis pathogen into the mammary gland induces local inflammatory reactions and the protective responses of the systemic immune system directly attracts/leads to an increase in milk somatic cell count (SCC), composed mainly of immune-related cells and exfoliated mammary gland epithelial cells. As such, the SCC has been generally regarded as an indirect measure of mammary gland health, whereby greater than 200,000 cells/mL is regarded as an indicator of an inflammatory response of the mammary gland in dairy cows (i.e. mastitis) [10–13]. According to the National Mastitis Council [14], SCC of normal milk is nearly always less than 200,000 cells/mL. Upon detecting the presence of mastitis pathogens, the immune system response which is according to pathogen type is under the regulation of genetic and epigenetic factors [15, 16].

DNA methylation, a well characterized epigenetic mechanism involved in the regulation of various biological processes, is reported to participate in the regulation of mammary gland health, including the occurrence and progression of mastitis [17–19]. DNA methylation alteration in response to mastitis caused by *Staphylococcus aureus* has been reported in peripheral blood lymphocytes [20] and mammary gland tissues [21]. Furthermore, the effect of DNA methylation on the transcription of genes in blood neutrophils and altered miRNA expression in response to *Escherichia coli (E. coli)*-induced mastitis has been reported [22]. In the liver of cows with *E. coli*-induced mastitis, demethylation of toll-like receptor 4 (*TLR4*) gene was observed [23].

These reports of altered DNA methylation patterns acknowledge the crucial roles of DNA methylation during an infection, and in particular during mastitis. At the same time, more questions abound about the underlying mechanisms, which are less evident, pointing to the importance of integrative investigations between the methylome and transcriptome as a creditable way to explore these open questions. According to the literature, no study has explored the potential regulatory roles of DNA methylation during mastitis caused by *S. uberis*. Therefore, this study aimed to construct the whole-genome-wide DNA methylation and gene expression patterns of milk somatic cells from cows with naturally occurring *S. uberis* subclinical mastitis as a way of uncovering the underlying DNA methylation regulatory mechanisms, and to identify candidate biomarkers with ability to discriminate *S. uberis*-infected cows from healthy cows.

## Methods

### Animal selection and sample collection

Holstein cows recruited for this study were from two commercial farms in Quebec, Canada, with a history of intramammary infections. The study period was from August 2020 to March, 2021. Cows were managed in a tie-stall system with saw dust as bedding. The milk SCC (an indirect measure of cow health) of 68 and 134 lactating cows in herd one and two, respectively, were monitored during the period of the experiment. The milk somatic cell counts of samples (10 mL/cow) collected once monthly from each cow were determined with Fossomatic flow cytometric cell counter (Foss, Hilleroed, Denmark) by Lactanet [24]. Thirty-four cows (9 from herd one and 25 from herd two) with ≥ 350,000 cells/mL (HSCC group) and twenty cows (10 from each herd) with ≤ 100,000 cells/mL (LSCC group) in their milk for three consecutive months were selected to test for the presence or absence of pathogens. Approximately 10 mL of milk per quarter was aseptically collected from each cow in the HSCC group or a composite milk sample (equal volume of milk from all four quarters) from each cow in the LSCC group, placed on ice and immediately (same day) sent to Biovet laboratories [25] for bacteriological examination. Bacteriological examination was for all non-fastidious bacteria and mesophilic microbes known to cause mastitis such as *Streptococcus* species, *Staphylococcus* species, *Nocardia* species, yeast species, *Klebsiella* species, *Escherichia* species, *Aerococcus* species, *Micrococcus* species, and many others. HSCC cows only positive to *S. uberis* (*n* = 5) were selected to constitute the test group while LSCC cows negative for all mastitis pathogens (*n* = 9) tested constituted the control group. Note that, cows positive to more than one pathogen were excluded. During a second visit, about 200 mL of milk was sampled from one infected quarter/cow in the HSCC group (only one quarter was sampled even if more than one quarter was positive to *S. uberis*) while a composite sample (200 mL milk, 50 mL/quarter) was obtained from each cow in the LSCC group. Since about 3 to 5 days elapsed between testing cows for the presence of pathogens and the second sampling visit, another bacteriological test was performed on the day of the second sampling to confirm the first bacteriological results. Only samples with consistent results and being in parity 1 to 3 and in middle to late lactation stage were kept for this study, including three *S. uberis*-positive quarters from three cows as test group and three *S. uberis*-negative cows as control group.

Following collection, milk samples were immediately transported to the laboratory on ice and milk somatic cells were isolated immediately upon getting to the laboratory. The milk somatic cells were isolated by low speed centrifuge (1500 × *g* for 15 min at 4 °C) followed by two times washing with phosphate buffered saline (PBS) (40 mL 1 × PBS added and centrifugation at 1500 × *g* for 15 min at 4 °C). Milk somatic cells were separated into two parts and stored at − 20 °C (for DNA isolation) or − 80 °C (for RNA isolation). The portion for RNA isolation was placed in TriZol reagent before storage.

### DNA isolation, library construction and DNA methylation sequencing

Genomic DNA was isolated using the DNeasy Blood and Tissue Kit (Qiagen Inc., Toronto, ON, Canada), and quantified using the Quant-iT™ PicoGreen® dsDNA Assay Kit (Life Technologies, Burlington, ON, Canada). Genomic DNA was used for whole genome DNA methylation sequencing library construction using the NEBNext® Enzymatic Methyl-seq Kit (New England BioLabs Ltd., Whitby, ON, Canada) [26], and quantified using the Kapa Illumina GA with Revised Primers-SYBR Fast Universal Kit (Kapa Biosystems Inc., Wilmington, MA, US). Average size fragment was determined using a LabChip GX (PerkinElmer Inc., Waltham, MA, US) instrument. The libraries were normalized and pooled in equimolar concentrations and then denatured in 0.05 mol/L NaOH and neutralized using HT1 buffer. The pool was loaded at 225 pmol/L on an Illumina NovaSeq S4 lane using Xp protocol as per the manufacturer’s recommendations. The run was performed for 2 × 100 cycles (paired-end mode). Library construction and sequencing were performed by Centre d’expertise et de services Génome Québec [27].

### RNA isolation, library preparation and sequencing

Total RNA was isolated from milk somatic cells with the RNeasy Mini Kit (Qiagen Inc., Toronto, ON, Canada) according to manufacturer’s protocol. Total RNA was quantified using Agilent Bioanalyzed 2100 (Agilent Technologies, Saint-Laurent, QC, Canada) and its integrity assessed on a LabChip GXII (PerkinElmer Inc., Waltham, MA, US) instrument. All RNA samples analyzed had RIN (RNA integrity number) values greater than 7. Ribosomal RNA was depleted from 125 ng of total RNA using QIAseq FastSelect Kit (Qiagen Inc.). cDNA synthesis was achieved with the NEBNext RNA First Strand Synthesis and NEBNext Ultra Directional RNA Second Strand Synthesis Modules (New England BioLabs). The remaining steps of library preparation were done using the NEBNext Ultra II DNA Library Prep Kit for Illumina (New England BioLabs). Adapters and PCR primers were purchased from New England BioLabs. Libraries were quantified using Kapa Illumina GA with Revised Primers-SYBR Fast Universal kit (Kapa Biosystems). Average size fragment was determined using a LabChip GXII (PerkinElmer) instrument.

The libraries were normalized and pooled in equimolar proportions and then denatured in 0.05 mol/L NaOH and neutralized using HT1 buffer. The pool was loaded at 200 pmol/L on an Illumina NovaSeq S4 lane using Xp protocol as per the manufacturer’s recommendations. The run was performed for 2 × 100 cycles (paired-end mode). A phiX library was used as a control and mixed with libraries at 1% level. Base calling was performed with RTA v3.4.4. Program bcl2fastq2 v2.20 was then used to demultiplex samples and generate fastq reads.

### Methylome data processing and global methylome comparison between *S. uberis*-positive and control groups

The raw DNA methylation sequencing data was processed using nf-core methylseq analysis pipeline [28]. The “EM Seq” trimming profile was selected to avoid potential bias towards non-methylation at the end of reads (8 bp) caused by end repairing. Sequence quality report was generated using FastQC (version 0.11.9) while adapter sequences and low-quality reads were trimmed with Trim Galore! (Version 0.6.5). The high-quality trimmed reads were merged and mapped to the bovine reference genome (ARS-UCD1.2) using bowtie2 under Bismark (version 0.22.3). Following alignment, Samtools (version 1.11) was used to merge, sort and remove duplicates, and generate BAM files for next-step analysis. Methylation calling was performed with bismark_methylation_extractor under Bismark. The methylation sites identified in all samples and with greater than 7 × coverage depth were retained for further processing.

The R package, Methylkit (version 3.12) [29] was used to detect differentially methylated cytosines (DMCs) and differentially methylated regions (DMRs) between *S. uberis*-positive and healthy control groups. Parity and lactation stage were set as batch factors to eliminate batch effects and to decrease random noise. DMC was defined as methylation sites with greater than 20% difference in methylation level between the two groups and *q* value < 0.05. DMR was scanned by a 1000-bp window size with 1000 bp step and filtered based on 20% methylation difference and having a *q* value < 0.05 and harboring ≥ 3 DMCs.

### Methylation haplotype block identification and comparison

Methylation haplotype blocks (MHBs) were detected using MONOD2 and following authors’ guidelines [30]. In brief, the clean DNA methylation sequencing reads of all samples were pulled together and then used to split the bovine reference genome (ARS-UCD1.2) into non-overlapping segments that were sequenceable and mappable. The methylation haplotypes were identified from mapped reads in each segment. The MHBs were identified based on methylation linkage disequilibrium which was calculated on the combined methylation haplotypes. MHBs were defined as the regions in which *r*^2^ value of any two adjacent CpG sites was ≥ 0.5, and MHBs containing at least 3 CpG sites were retained.

Next, methylated haplotype load (MHL), which is the normalized methylation level of methylated haplotypes at different lengths, was calculated for all MHBs in each sample. Differential MHBs were firstly filtered by selecting those having more that 20% difference in MHL between groups. Then, two-tailed Student’s *t*-test was used for differential MHL analysis between *S. uberis*-positive and healthy control groups, to detect significant differential MHBs (dMHBs). Benjamini and Hochberg false discovery rate (FDR) correction [31] was used to adjust *P* values and FDR < 0.05 was considered significant.

### Identification of differentially expressed (DE) genes

The nf-core bioinformatics pipeline for RNA-Seq data [28] was used to analyze RNA sequencing reads. Briefly, adapters and low-quality reads (quality score < 30) were removed using Trim Galoire! [32]. Clean reads were aligned to ARS-UCD1.2 (cow reference genome) using STAR [33] and then the downstream BAM-level quantification was performed with Salmon [34], followed by deduplication with UMI-tools [35]. Differential expression of genes between *S. uberis*-positive and healthy control groups was analyzed with DESeq2 (version 1.34.0) [36]. Parity and lactation stages of cows were included as batch factors during analysis. Significant differentially expressed (DE) genes were defined as having a Benjaminin and Hockberg corrected FDR < 0.05 [31] and |log_2_ fold change| (|log_2_FC|) ≥ 1.

### DNA methylation and gene expression integration analysis

The global correlations between DNA methylation alterations and gene expression changes were determined with MethGET program [37]. Briefly, the CGmap file of CpG sites converted from methylation coverage report files generated by Bismark, normalized gene expression file generated by DESeq2, and gene annotation GTF file converted from the latest RefSeq assembly accession (GCF_002263795.1) were used as the input files for MethGET. Firstly, the annotation GTF file was used to identify different genomic regions (promoters, gene bodies, exons and introns) followed by calculation of the methylation levels of each gene at different genomic regions, by averaging the methylation levels of all CpG sites in the corresponding regions. “Single-methylome analyses” option in MetGet was used to correlate the methylome and transcriptome of individual samples. This was done for each sample separately to investigate the possible association between the DNA methylation status and gene expression level. Besides, the correlation between the DNA methylation alterations and the changes in gene expression between *S. uberis*-positive and healthy control groups was measured by genome wide Person’s correlation analysis. Moreover, Gaussian Mixture Model (GMM) from the scikit-learn package in python was used to identify genes with significant changes in DNA methylation levels and/or gene expression levels (*P*-value < 0.001), and here referred to as MetGDE genes [38]. GMM was also used to obtain MetGDE genes based on the methylation status of the promoter, gene body, exons and introns, separately, to understand their possible effects on the expression of genes.

### Identification of biomarkers discriminating *S. uberis* infected cows from healthy cows

The core DIABLO method from R package, mixOmics [39], was used to identify highly correlated DNA methylome and transcriptome changes capable of discriminating *S. uberis*-infected cows from healthy cows. DIABLO is an improved extension of the multivariate methodology, Generalised Canonical Correlation Analysis (GCCA), but it generalizes Projection to Latent Structure (PLS) for matching multiple datasets and the sparse GCCA method [40]. For this study, the input data included: (1) the top 25% most variable DE genes, (2) the top 25% most variable DMCs located in DE genes and their 2000 bp up- and down-stream regions, and (3) all identified dMHBs. The key parameters of DIABLO implemented were chosen according to the authors’ recommendations [39]. The full weighted design was used to get the trade-off between maximizing correlation between input datasets and the discrimination of selected biomarkers. The number of components was set as 1 (one less than number of groups, which is 2 groups − 1 = 1) in this study, which is sufficient to get the best discriminable performance according to the authors’ recommendation [39]. The first-rank number of candidate biomarkers were determined to achieve the minimum number of signatures with best performance by 3-fold cross-validation repeated 10 times from a grid of 5 to 50.

### Functional enrichment of select genes and other information

The functional enrichment analysis of select genes (genes harboring DMCs, genes overlapping with dMHBs, DE genes, and MetGDE genes), was performed with DAVID Bioinformatics Resources v6.8 [41] and visualized with R packages ggplot2 [42] and GO plot [43]. FDR < 0.05 was used as threshold to define significantly enriched Gene Ontology (GO) terms and KEGG (Kyoto Encyclopedia of Genes and Genomes) pathways.

The genome structure annotation files for genes, repeats and CpG island (CGI) of the bovine reference genome (ARS-UCD1.2) were download from UCSC Table browser [44]. The promoter (upstream) was defined as the 2 kb region upstream of the transcription start site (TSS). “RepeatMasker” was chosen as the track for repeats. According to genomic location and relative to CGI, CGI shores left and right were defined as the 2 kb region upstream of the CGI or the 2 kb region downstream of the CGI, respectively, and CGI shelves left and right were defined as the 2 kb region upstream of the left CGI shore or the 2 kb region downstream of the right CGI shore, respectively. Meanwhile, CpG desserts (left or right) were the regions more than 4 kb upstream or downstream of CGIs, respectively. The R package, annotatr (version 3.12), was used to detect the enrichment categories of DMCs, DMRs and MHBs in these genomic regions.

### Validation of RNA-sequencing by real-time qPCR

The expression levels of 9 genes, including 6 DE and 3 non-DE genes, were evaluated by real-time qPCR as a validation of the RNA-sequencing data (Table S1). Primers for selected genes were designed with Primer-BLAST [45]. Firstly, 1 μg total RNA (per sample) of the same RNA used for RNA-sequencing was reverse transcribed into cDNA using SuperScript™ IV VILO™ Master Mix (Invitrogen, Waltham, Massachusetts, USA). The cDNA was then diluted 1:15, and used for gene specific qPCR amplification. The 10 μL qPCR reaction mix consisted of 1 μL (80 ng) cDNA, 5 μL PowerTrack™ SYBP™ Green Master Mix (Applied Biosystems, Waltham, Massachusetts, USA), 0.25 μL Yellow sample buffer, 0.5 μL each of forward and reverse primers (500 nmol/L each) and 2.75 μL nuclease-free water. The real-time amplification was performed on a StepOnePlus™ instrument (Applied Biosystems) using a fast cycling mode. The fast cycling mode started with 2 min enzyme activation at 95 °C, followed by 40 cycles of denaturation at 95 °C for 5 s and extension at 60 °C for 30 s. *ACTB* (β-action) was used as the reference gene to normalize the gene expression, and the relative expression value of genes were calculated by using 2^-∆∆Ct^ method [46].

## Results

### Global DNA methylation level trends of milk somatic cells

**Fig. 1:**
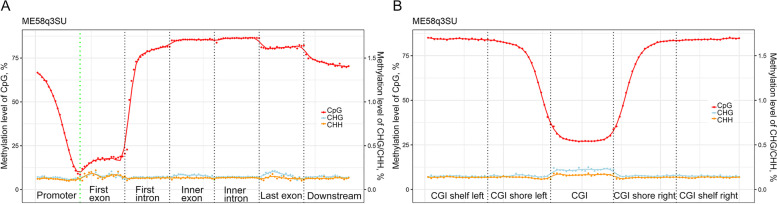
DNA methylation level trends among gene features (**A**) and in CpG island (CGI) (**B**). The promoter is the 2 kb region upstream of the transcription start site. **A** and **B** plots are exampled by one sample (ME58q3SU). The downstream is the 2 kb region downstream of the transcription termination site. CGI shores are the 2 kb regions flanking the CGI. CGI shelf left is the 2 kb region upstream of CGI shore left while CGI shore right is the 2 kb region downstream of CGI shore right. The plots of all samples are shown in additional Fig. S1

On average, 11.7 billion cytosine sites (based on an average of ≥ 20.2 reads coverage per site) per sample were detected in the context of CpG, CHG and CHH to build up the genome-wide DNA methylation pattern of bovine milk somatic cells (Table S2a-b). To ensure good quality of the data and high confidence methylation levels, only cytosines with at least 7 reads coverage in all samples were kept for further processing. In addition, cytosines with extremely high coverage (top 0.1%) were removed to eliminate possible bias. The methylation levels of milk somatic cells from *S. uberis*-infected and healthy cows showed relatively consistent distribution trends (Fig. S1A). Similar with other mammals, CpG sites was the main form of methylation detected. The methylation levels of the abundant CpG sites were higher than 76% (Fig. S1A and Table S2a). Meanwhile, the CHG and CHH sites rarely underwent methylation, with mean methylation levels of 0.20% and 0.12%, respectively (Fig. S1A and Table S2a). The methylation level trends among gene features are shown in Fig. 1A and Fig. S1B. The methylation level of CpG sites showed a sharp decrease from approximately 70% to 10% in the promoter region, remained extremely low in the first exon (~ 20%), then increased in the first intron up to > 75% and remained at high levels (higher than 80%) in inner exons and introns, but decreased slightly in the last exon and decreased further in downstream region (Fig. 1A). CHG and CHH sites had very low methylation levels, around 0.1%–0.2% in the different gene features without big volatility (Fig. 1A and Fig. S1B). The methylation level of CHG sites were higher in exons, showing small peaks in the first, inner and last exons. While the CHH sites only showed higher methylation in the first exon. Considering the relative position to CGIs, the methylation level of CpG sites were low in CGIs (~ 25%), increased gradually until ~ 80% in CGI shores and remained high in CGI shelves (Fig. 1B, Fig. S1C). Interestingly, the methylation levels of CHG and CHH sites were higher in CGIs (about 0.25% and 0.18%, respectively) but remained low (~ 0.14%) in CGI shores and shelves, which is an opposite trend compared with CpG site (Fig. 1B, Fig. S1C).

### DNA methylation comparison between *S. uberis*-positive and healthy groups

**Fig. 2:**
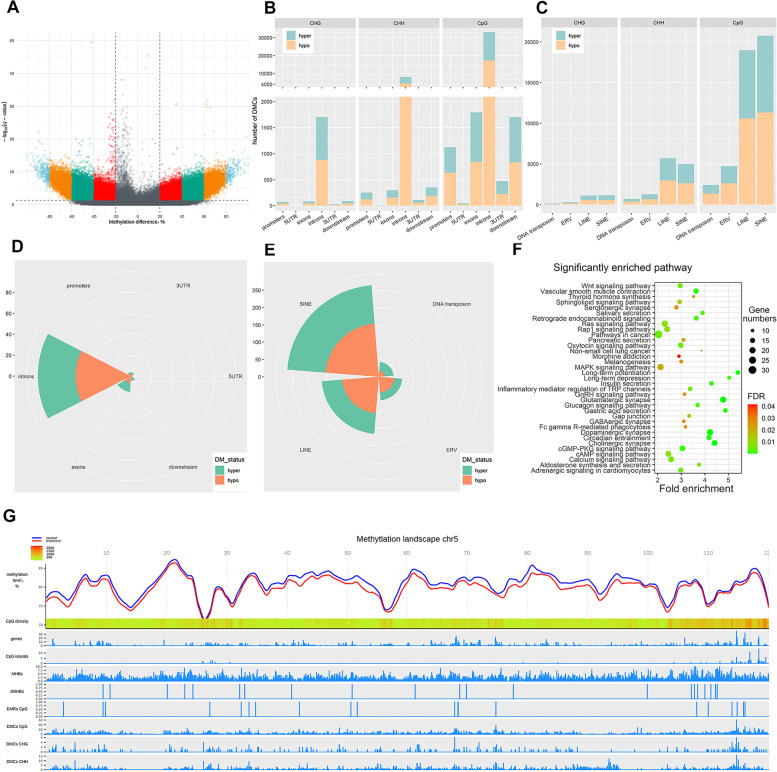
DNA methylation alterations between *S. uberis*-positive and healthy control groups. **A** Volcano plot showing DMCs in the context of CpG. Annotation of DMCs in gene features (**B**) and repeat elements (**C**). The annotation of DMRs in gene features (**D**) and repeat elements (**E**). **F** KEGG pathways significantly enriched by genes harboring ≥ 15 DMCs. **G** Landscape showing the DNA methylation status of chromosome 5. A window of 50 kb was used to count the information per track

A total of 132,237, 7412 and 34,693 DMCs in the context of CpG, CHG and CHH were identified between *S. uberis*-positive and control groups, accounting for 0.60%, 0.0069% and 0.0095% of total CpG, CHG and CHH sites, respectively (Fig. 2A, Fig. S2A-B, Table S3a-c). The hypo-DMCs (CpG: 72,992, CHG: 3822, CHH: 18,464) accounted for 55.20%, 51.57% and 53.22% of the total DMCs in the CpG, CHG and CHH contexts, respectively and were slightly higher than the number of hyper-DMCs (CpG: 59,245, CHG: 3590, CHH: 16,229), respectively. The methylation level differences in majority of sites in CpG context were between 20% and 60% (Fig. S2C).

The annotation to functional genic features revealed that most DMCs were located in intergenic regions, followed by repeat elements and genes (Table S4a). DMCs in the three contexts (CpG, CHG and CHH) were similarly distributed in these genomic regions (intergenic regions, repeat elements and genes). Within genes and related regulatory features, approximately 90% of DMCs in the three contexts were located in introns followed by exons while the 3’UTR and downstream regions had more DMCs than 5’UTRs and promoters, respectively (Fig. 2B). Most DMCs in repeat elements were in non-long terminal repeats (non-LTRs), including long interspersed nuclear element (LINE) and short interspersed nuclear element (SINE), followed by endogenous retrovirus (ERV) and DNA transposons (Fig. 2C). Approximately 80% of DMCs in repeat elements were located in intergenic regions while the remaining 20% were mostly located in intronic regions of genes (Fig. S3A-C). The annotation in relation to CGIs indicated that most DMCs were located in CGI deserts (Fig. S3D). CGI-shores had more DMCs than CGI shelves, while CGIs had the lest DMCs. Besides, the majority of DMCs in these genic features had 20%–60% difference in methylation levels between *S. uberis*-positive and control group (Fig. S3E-F).

A total of 316 DMRs were identified based on CpG sites, including 129 hyper-DMRs and 187 hypo-DMRs (Table S5a), while no DMR was found in CHG or CHH contexts. The annotation of DMRs revealed that the majority of DMRs were located in CpG deserts and in intronic regions of genes (Fig. 2D, Table S5c). In addition, most DMRs were found within transposons, including SINE, LINE and ERV, and more than half of these DMRs in repeat elements were hypo-methylated (Fig. 2E).

Chromosomal distribution of DNA methylation changes indicated that about 99% of DMCs and DMRs were annotated to chromosomes while 1% were annotated to unplaced genomic scaffolds (Table S4b, Table S5b). Chromosome 1 (Chr1) had the most DMCs (*n* = 9637, including 7221 CpG-DMCs, 344 CHG-DMCs and 2072 CHH-DMCs), followed by Chr5 (*n* = 9393; 7746 CpG-DMCs, 320 CHG-DMCs and 1327 CHH-DMCs) and Chr14 (*n* = 8855; 6543 CpG-DMCs, 425 CHG-DMCs and 1887 CHH-DMCs) (Table S4b, Fig. S4A). Manhattan plots of chromosomal distribution of DMCs showed higher density of CpG-DMCs on all chromosomes (Fig. S4B) when compared to lower densities of CHG-DMCs (Fig. S4C) and CHH-DMCs (Fig. S4D). Furthermore, chromosomal ends where characterized by a high density of genes, CpG-DMCs and CGIs (Fig. 2G, Fig. S4B), suggesting important regulatory roles of DNA methylation at the ends of chromosome. Overall, the average methylation level of *S. uberis*-positive group was lower than that of the healthy control group (Fig. 2G, Fig. S4E).

A total of 7114 genes were found to harbor DMCs (CpG, CHG or CHH) in their gene body and/or promoter regions, while 76 genes were found to overlapped with at least one DMR (Table S6). Approximately 34.66% (*n* = 2466) of genes harboring ≥ 5 DMCs and the top 10% of genes (*n* = 765) harboring ≥ 15 DMCs in their gene body and promoter (Table S6a) were submitted for functional annotation analysis by DAVID, to investigate the potential biological roles of DNA methylation changes. Two cellular component (CC) GO terms, two molecular function (MF) GO terms and 35 KEGG pathways were significantly enriched (Table S7a). As shown by the enriched KEGG pathways (Fig. 2F), DNA methylation changes affected biological functions reflected by pathways such as glutamatergic synapse (bta04724, FDR = 4.92 × 10^−6^), dopaminergic synapse (bta04728, FDR = 1.94 × 10^−5^) and cholinergic synapse (bta04725, FDR = 3.79 × 10^−5^), as well as disease and immune pathways (inflammatory mediator regulation of TRP channels (bta04750, FDR = 0.005), long-term depression (bta04730, FDR = 0.01), and pathways in cancer (bta05200, FDR = 0.005), etc.). Since promoter region DMC is of particular interest, we also submitted genes harboring ≥ 3 DMCs in their promoter regions (*n* = 278), but no GO term or pathway were enriched.

### DNA methylation haplotype blocks (MHBs) responding to *S. uberis* subclinical mastitis

**Fig. 3:**
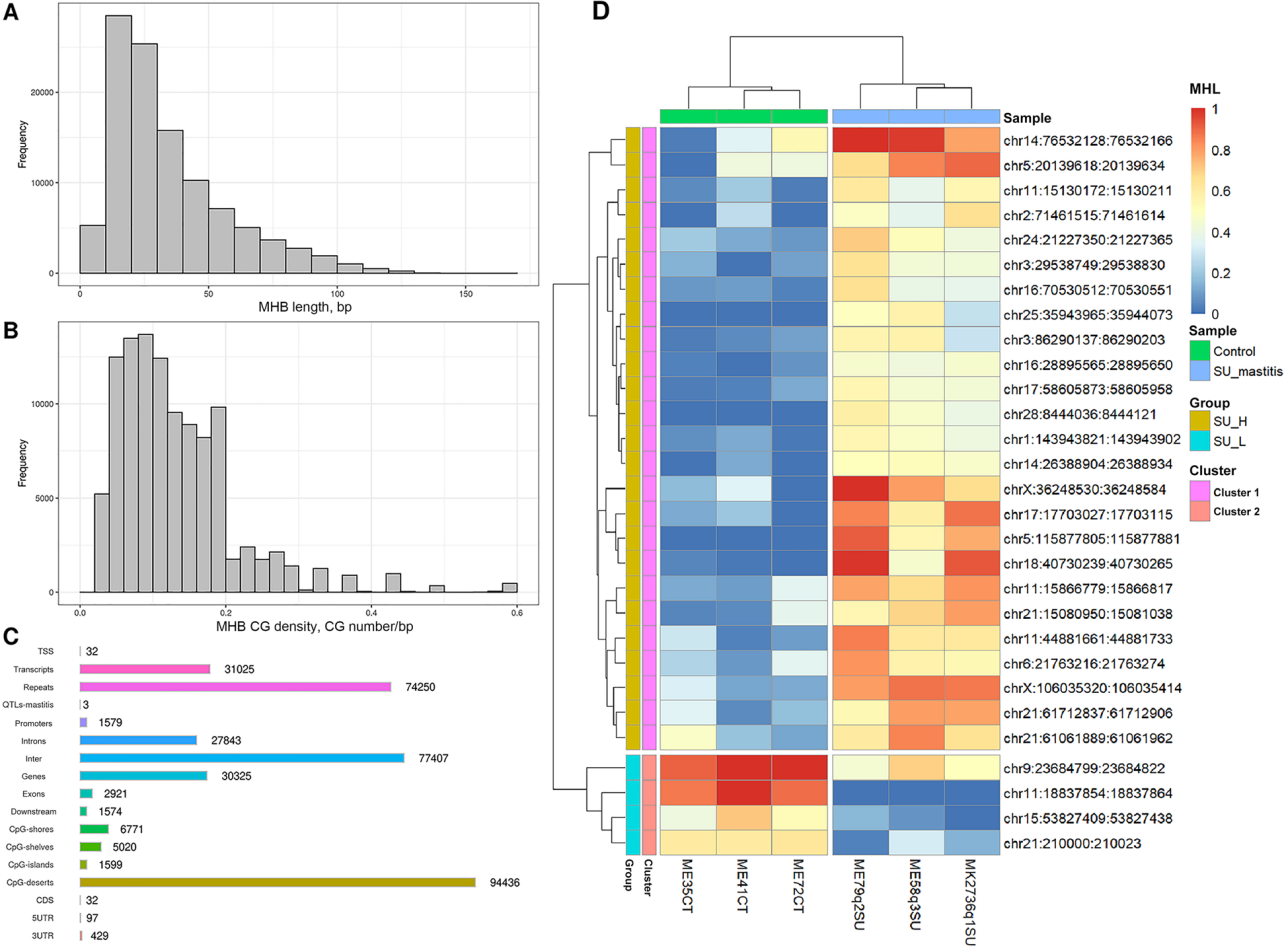
Methylation haplotype blocks (MHBs). **A** length distribution of MHBs. **B** CpG density distribution of MHBs. **C** Annotation of MHBs in different genetic features. **D** Heatmap showing the methylation status of the most variable MHBs with more than 40% difference in methylation haplotype load (MHL) between *S. uberis*-positive and healthy control groups

Using the methylome data of all samples, the bovine genome was partitioned into blocks of tightly coupled CpG methylation sites (*r*^2^ cutoff of 0.5), referred to as MHBs. A total of 101,612 MHBs were identified with a minimum of 3 CpG sites per block and average CpG density of 0.13 CpG sites/bp (Fig. 3A-B). The average size of MHBs was 35 bp (range from 5 to 164 bps). A greater number of CpG sites inside MHBs were nearly, perfectly coupled (*r*^2^ ~ 1.0). The MHBs partially or perfectly overlapped with different types of known genomic elements (Fig. 3C). For example, 77,407 (76.18%) MHBs are located in intergenic regions, while 8379 (30.53%) are located within transcripts. Out of the 30,325 MHBs located within genes, 27,843 are found within introns, which is about 10-fold more than in exons (12,921). Moreover, 1579 MHBs were overlapped with promoter regions, including 32 MHBs spanning TSSs. Interestingly, 74,250 MHBs were located in repeat elements. Further annotation of MHBs according to genomic CpG density revealed a majority of MHBs in CpG deserts (94,436), followed by CGI shores (6771), CGIs (1599) and CGI shelves (5020).

Methylation haplotype load (MHL) was calculated for all MHBs in each sample to enable quantitative analysis of MHB methylation patterns across samples. A total of 2379 MHBs had more than 20% difference in MHL between *S. uberis*-positive and control group. Among them, the MHL of 451 MHBs were significantly different between the two groups (FDR < 0.05), and were referred to as differential MHBs (dMHBs) (Table S8a). The dMHBs with the most significant difference in MHL (> 40%) were used to plot a heatmap, which showed clear segregation between *S. uberis*-positive and control groups (Fig. 3D). More than 80% of all dMHBs had higher methylation levels in *S. uberis*-positive group (*n* = 367), and were enriched in two CC-GO terms (cytosol (GO:0005829) and cytoplasm (GO:0005737)) (Table S8b). The methylation levels of dMHBs in some cases were very high in *S. uberis*-positive group while being very low or absent in control group and vice versa. For example, a dMHB in Chr5 (chr5:115877805–115877881) had very high methylation level in *S. uberis*-positive group (average MHL = 0.74) but unmethylated in healthy control group (MHL = 0). On the contrary, a dMHB in the intron of *CRIM1* (chr11:18837854–18837864) was nearly completely methylated in the control group (average MHL = 0.91) but unmethylated in *S. uberis*-positive group (MHL = 0). In addition, 182 dMHBs were found within genes, and 9 dMHBs within the promoter regions of *ATP6V1E2, CBX6, ARHGAP15, CRYGS, CELA2A, RFTN2, TFEB, DOK3 and CCDC115*. A dMHB in Chr2 (chr2:86177974–86178029) with a higher MHL in *S. uberis*-positive group was found in the promoter region of *RFTN2*, a gene detected as DE with a 9.6 times higher expression level in *S. uberis*-positive group (Table S8a, Table S9a). Interestingly, a dMHB (chr7:39010791–39010831) harboring 3 DMCs is overlapped with the promoter of *DOK3* as well as the downstream region of *DDX41*. Similarly, a dMHB on Chr2 (chr2:1320056–1320082) is overlapped with the promoter region of *CCDC115* and the intron of *IMP4*.

### Differentially expressed (DE) genes in response to *S. uberis*

**Fig. 4:**
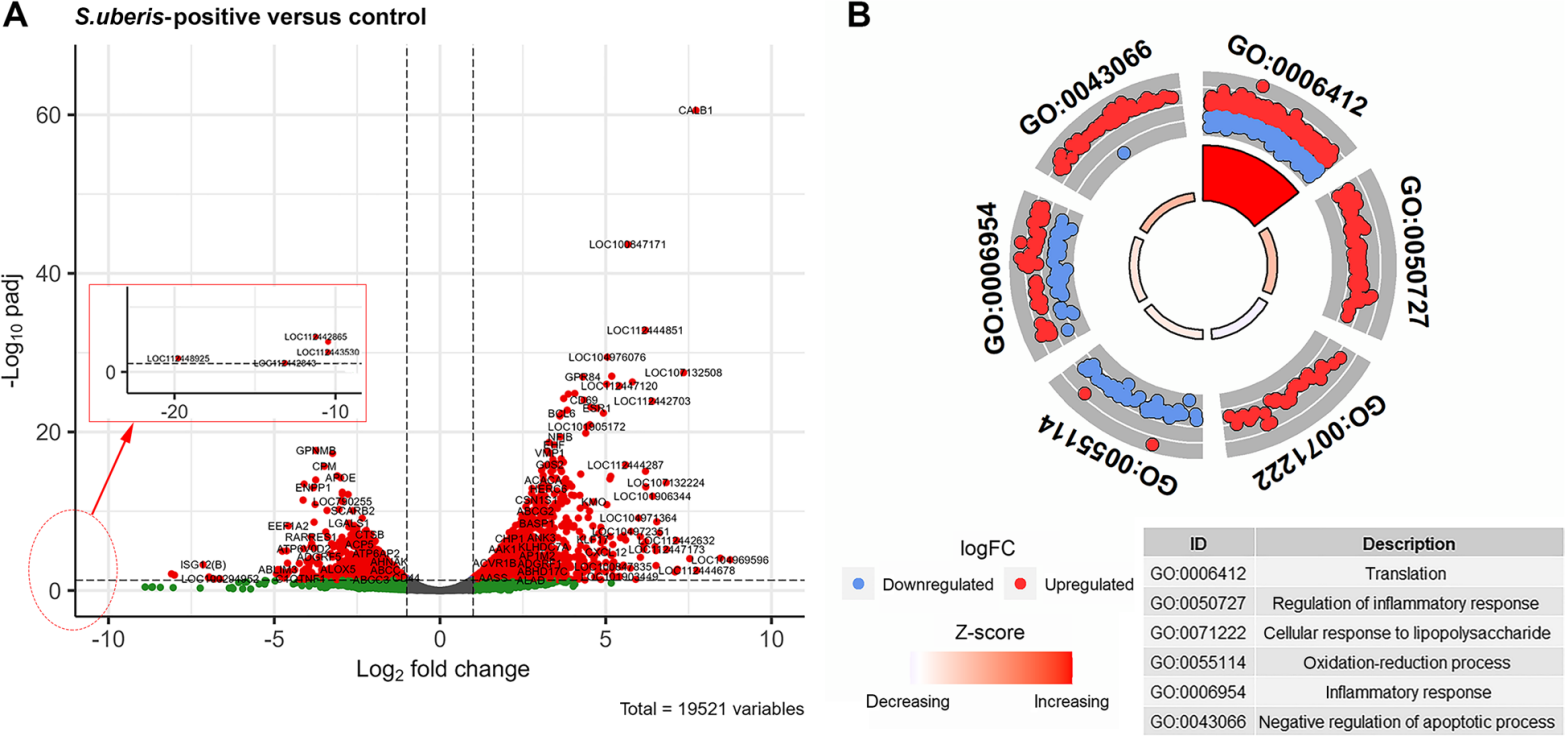
Differentially expressed (DE) genes between *S. uberis*-positive and healthy control groups. **A** Volcano plot showing expression changes of DE genes. **B** Biological process gene ontology terms significantly enriched by DE genes

RNA-seq technology was used to detect the genome-wide gene expression in milk somatic cells in response to *S. uberis* subclinical mastitis. A total of 2130 DE genes were identified (FDR < 0.05 and |log_2_FC| > 1) (Table S9a, Fig. 4A), including 1378 up-regulated and 752 down-regulated DE genes in response to *S. uberis* subclinical mastitis. Functional annotation analysis of the DE genes resulted in 22 enriched GO terms, including 6 biological process (BP) terms, 13 CC terms and 3 MF terms (Table S9b). It is worthy to note that, half of the enriched BP terms were related to immune responses, including regulation of inflammatory response (GO:0050727, FDR = 0.01), cellular response to lipopolysaccharide (GO:0071222, FDR = 0.01) and inflammatory response (GO:0006954, FDR = 0.02) (Fig. 4B). In addition, 19 KEGG pathways were enriched (Table S9b), including 8 pathways related to disease or the immune response, such as leukocyte transendothelial migration (bta04670, FDR = 0.03), chemokine signaling pathway (bta04062, FDR = 0.038) and biosynthesis of antibiotics (bta01130, FDR = 0.038). Ribosome (bta0301, FDR = 2.09 × 10^−19^) was the most significantly enriched pathway, followed by lysosome (bta04142, FDR = 1.67 × 10^−13^) and osteoclast differentiation (bta04380, FDR = 1.32 × 10^−8^).

**Fig. 5:**
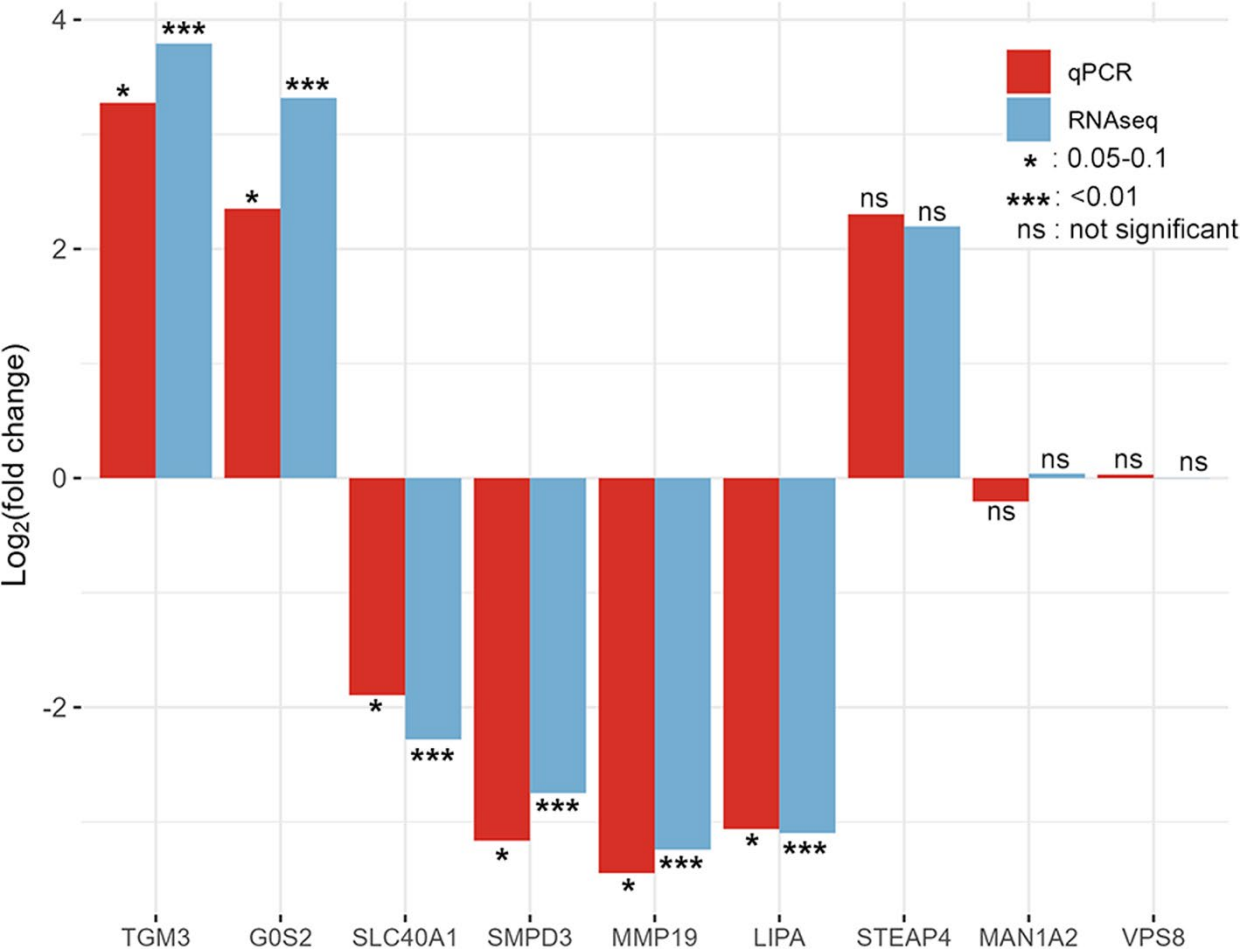
Expression results of select genes by real-time qPCR compared with results of RNA sequencing

The real time qPCR expression results of two up-regulated, four down-regulated DE genes and three non-DE genes randomly selected for verifying RNA-seq data, were similar with the expression results obtained by RNA-sequencing. The three non-DE genes identified by RNA-sequencing were also non-DE by real-time qPCR (Fig. 5). The relative expression value (log_2_FC) of all DE genes were similar by both RNA-sequencing and real-time qPCR methods (Fig. 5).

### Relationship between DNA methylation and gene expression during *S. uberis* mastitis

**Fig. 6:**
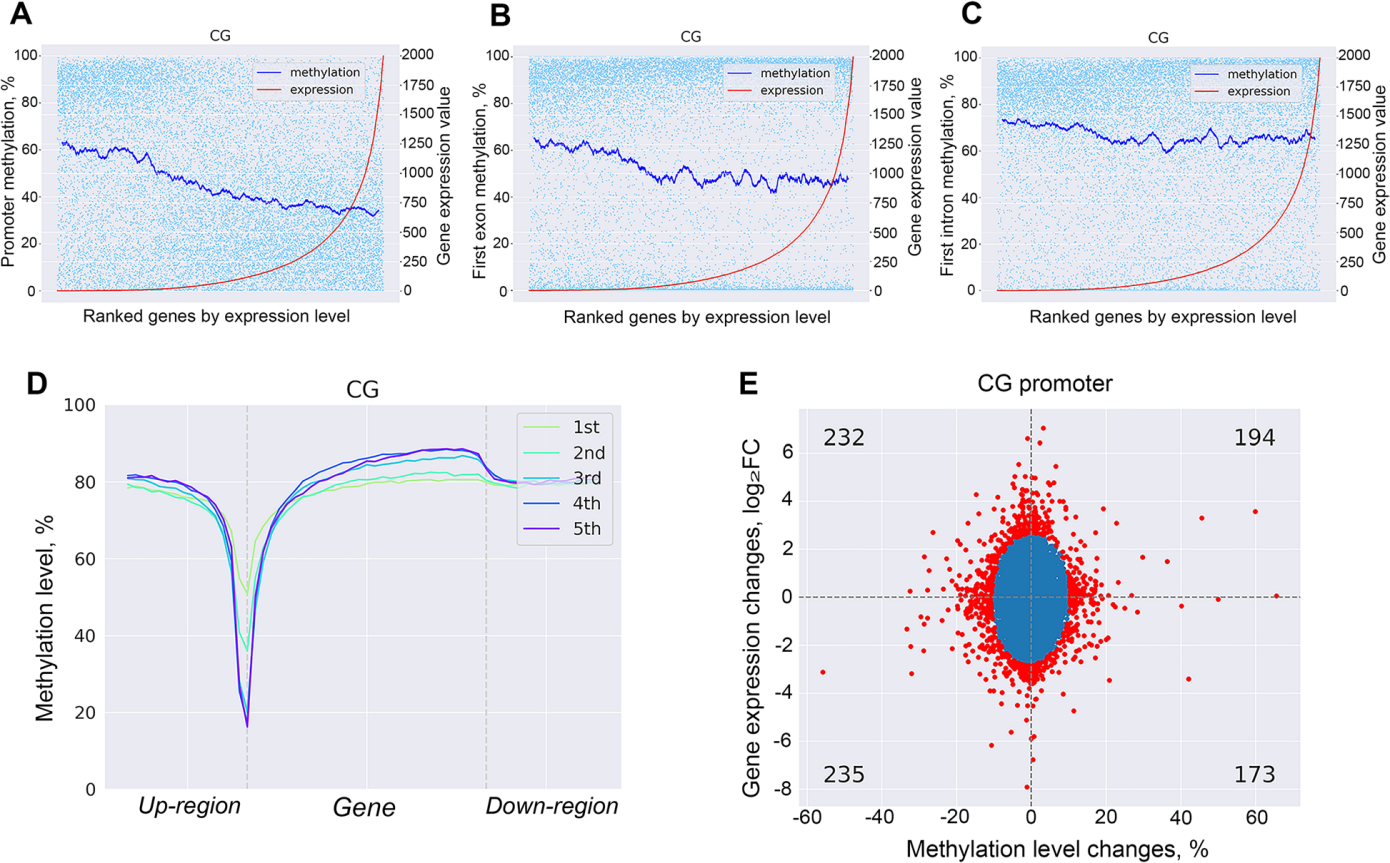
Association between DNA methylation changes and gene expression levels. The scatterplot and fitting curves of DNA methylation and relative gene expression: **A** promoter methylation, **B** first exon methylation and **C** first intron methylation. **D** The methylation level trends according to gene group with differential expression level around gene body regions. Up-region represents the upstream region half the length of corresponding gene body, same for down-region. **E** Scatter plot showing changes of DNA methylation levels in promoter regions and gene expression changes. Red dots signify promoter-MetGDE genes

Overall, the DNA methylation at promoter regions and first exons showed significant negative correlations with gene expression (*P* < 5 × 10^−8^), and as the DNA methylation level decreases, the gene expression level increases (Table S10a, Fig. 6A-B, Fig. S5A-C). DNA methylation level at first intron showed negative but weaker correlation with gene expression level (Fig. 6C, Table S10a). No significant correlation was found between gene expression and methylation in gene body, introns or exons (R: ~ 0, *P* > 5 × 10^−8^) (Table S10a). In addition, the methylation level in promoter regions were generally lower than in gene body, where methylation level was relatively stable and fluctuated between 70% and 80% with increasing gene expression (Fig. S5D-F). The DNA methylation was then profiled across different gene expression groups (*n* = 5), revealing similar general methylation trends (Fig. 1A, Fig. 6D). Moreover, downward trends in methylation levels (steeper downward trend for highly expressed genes (4^th^ and 5^th^ quantiles)) were observed at the upstream regions of genes, with a reverse trend (gradual increase) from the TSS and reaching about 80% methylation levels at the first intron (Fig. 1A, Fig. 6D). The methylation at gene body of genes with higher expression levels (3–5^th^ quantile) were higher than in genes with lower expression levels (1–2^nd^ quantiles) (Fig. 6D).

The changes in gene expression and alterations of DNA methylation at the region of promoter, gene body, introns or exons were next compared between *S. uberis*-positive and control groups, respectively. The correlation between methylation level alterations and gene expression changes were significant but weak at the promoter (*R* = 0.055, *P* = 1.93 × 10^−14^), gene body (*R* = 0.053, *P* = 8.84 × 10^−14^), introns (*R* = 0.062, *P* = 3.3210^−17^) and exons (*R* = 0.036, *P* = 5.03 × 10^−7^). A total of 834 genes, defined as promoter-MetGDE genes, were identified by GMM as showing significant changes in DNA methylation at their promoter regions and/or gene expression levels (*P* < 0.001) (Fig. 6E). Meanwhile, 577 gene body-, 597 introns- and 816 exons-MetGDE genes were identified showing significant gene expression changes and/or altered methylation levels (*P* < 0.001) (Fig. S5G-I). Taken together, a total of 1623 MetGDE genes were found showing significant changes in gene expression and/or DNA methylation status at one or more regions (promoter, gene body, introns and/or exons) (Table S10b). In addition, 234 MetGDE genes with significant changes in gene expression had significant DNA methylation changes in all four studied gene features (promoter, gene body, introns and exons) (Fig. S5J). Besides, 339 MetGDE genes were found to harbor DMCs, including 56 MetGDE genes with ≥ 10 DMCs. *PRKG1* harbored the most DMCs (*n* = 101), followed by *AP3B1*(*n* = 81), *WDFY4* (*n* = 68), *SAMD12* (*n* = 68), *DPP10* (*n* = 63) and *SLC25A21* (*n* = 52), etc. (Table S10c). Six DMCs at the promoter region of *SLC25A21* showed decreased methylation levels (methylation difference = − 28.59%) and higher *SLC25A21* expression level (log_2_FC = 1.69) in *S. uberis*-positive group. Similarly, *PLCL1* with − 22.064% difference in promoter methylation level and changed expression level (log_2_FC = 1.60) harbored 31 DMCs in its gene body.

### Functional annotation of MetGDE genes

**Fig. 7:**
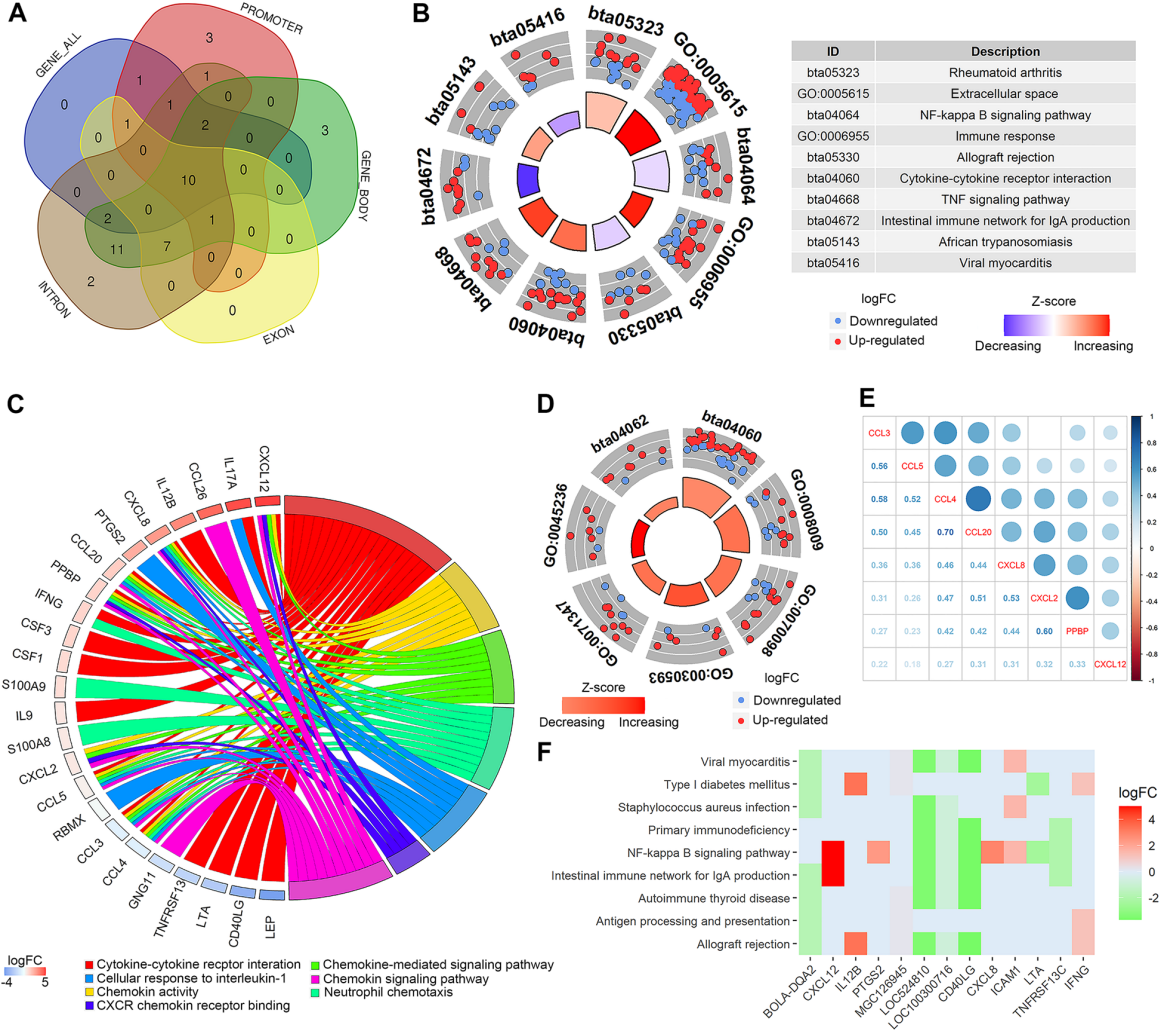
Functional enrichment of methylated and differentially expressed (MetGDE) genes. **A** The numbers of shared and unique functional annotations (GO terms and KEGG pathways) all MetGDE genes, promoter-, gene body-, intron- and exon- MetGDE genes. **B** Ten functional annotations commonly enriched by all five list of MetGDE gens. **C** and **D** A cluster of functional annotations related to cytokine activities. **E** The correlation between genes enriched in multiple GO terms and KEGG pathways related to cytokine activities. **F** A cluster of immune-related functional annotations and the enriched MetGDE genes

The possible biological effects of all the differential or MetGDE genes (*n* = 1623) selected by MethGET were investigated with DAVID bioinformatics resources resulting in significant enriched disease- and immune-related biological processes GO terms and pathways (one BP-GO term, 2 CC-GO terms and 14 KEGG pathways, FDR < 0.05) (Table S11a). Then, promoter-, gene body-, introns- and exons-MetGDE genes (*n* = 834, 577, 597 and 816, respectively) were each submitted to DAVID, resulting in 20, 36, 19 and 37 significant functional annotations, respectively (Table S11b-e). As shown in Fig. 7A, the five lists of MetGDE genes were enriched in similar biological processes, including 8 and 2 commonly enriched pathways and GO terms, respectively, with immune related functions (Table S11f, Fig. 7B). For example, extracellular space (GO:0005615) and immune response (GO:0006955) BP-GO terms, and TNF signaling pathway (bta04668) and Cytokine-cytokine receptor interaction (bta04060) pathways were more likely to be increased/activated (positive Z-score) while intestinal immune network for IgA production pathway was likely decreased (negative Z-score) during the host response to *S. uberis* subclinical mastitis (Fig. 7B). In addition, the significantly enriched annotations were clustered by similar biological effects, further suggesting possible roles of MetGDE genes on immune related processes (Table S12). For instance, a group of annotations related to cytokine processes and activities were clustered together, including Cytokine-cytokine receptor interaction (bta040600), chemokine activity (GO:0008009), chemokine-mediated signaling pathways (GO:0070098), cellular response to interleukin-1 (GO:0071347) and Chemokine signaling pathway (bta04062), etc., and most likely to have increased activities (positive Z-score) during *S. uberis* infection (Table S12b-e and Fig. 7C-D). Interestingly, some genes were commonly enriched in multiple pathways in this cluster, such as *CXCL12*, *CXCL8*, *CCL20*, *PPBP*, *CXCL2*, *CCL3*, *CCL4* and *CCL5* (Fig. 7C). These genes are related to each other and play key roles in the biological processes related to chemokine activities (Fig. 7E). Moreover, another cluster of annotations was related to diseases, such as Primary immunodeficiency (bta05340), Allograft rejection (bta05330) and Autoimmune thyroid disease (bta05320), and immune response pathways, represented by Nuclear factor-kappa B (NF-κB) signaling pathway (bta04064), Antigen processing and presentation (bta04612) and Intestinal immune network for IgA production (bta04672) (Fig. 7F). This cluster of annotation also revealed some commonly enriched MetGDE genes, including *CD40LG*, *LOC524810*, *BOLA-DQA2*, *LOC100300716*, *MGC126945*, *IFNG* and *ICAM1*.

### Candidate biomarkers distinguishing *S. uberis*-positive cows from healthy cows

**Fig. 8:**
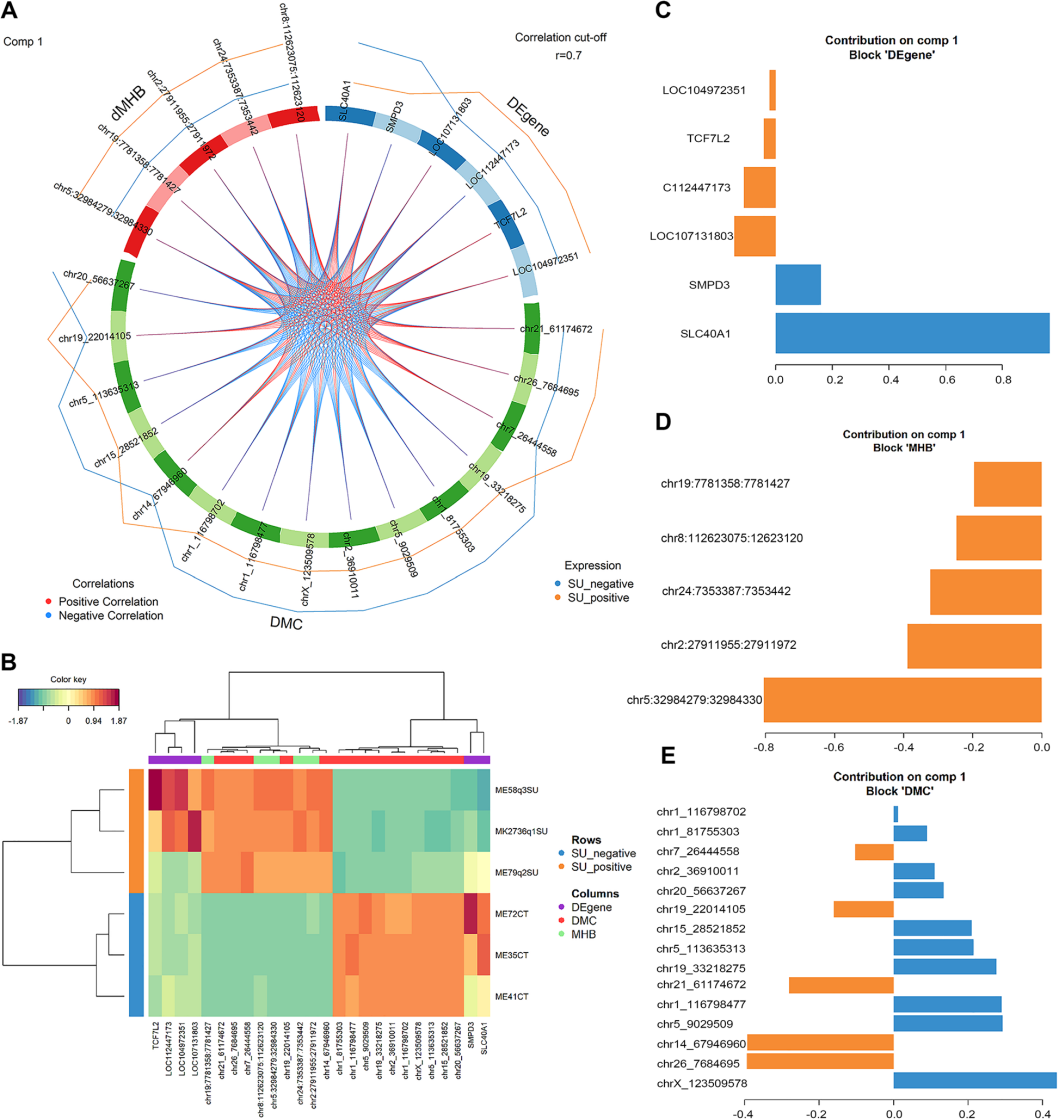
Candidate discriminant biomarkers of *S. uberis*-positive cows and healthy cows. (**A**) A circus plot showing the correlation between candidate biomarkers, including 6 DE genes (blue), 5 dMHBs (red), and 15 DMCs (in CpG context) (green). The external lines display the relative expression/methylation levels of selected candidates with respect to each outcome category. The yellow and blue lines represent the gene expression/methylation levels of *S. uberis*-positive and health control groups respectively, and the outer line represents the higher level. (**B**) Cluster of candidate biomarkers. Samples are represented in rows, while candidate biomarkers are represented in columns. (**C-E**) The loading plots of DE genes (**C**), dMHBs (**D**), and DMCs (CpG context) (**E**). The color of bar represents the group where the mean expression/methylation level is maximal. Orange and blue represent *S. uberis*-positive and healthy groups, respectively. The *x*-axis represents the loading weights, the negative value (orange bar) means the corresponding biomarker had higher expression/methylation level in *S. uberis*-positive group, while the positive value with blue bar means higher expression/methylation level in healthy control group

Application of DIABLO method to dMHBs, most variable DE genes and DMCs in the context of CpG, revealed a total of 26 candidate biomarkers of *S. uberis* subclinical mastitis, including 6 DE genes, 15 DMCs and 5 dMHBs (Tables 1 and 2). The 26 candidate biomarkers showed strong correlations with each other (|cor| > 0.7, Fig. 8A, Table S13). A heatmap of the biomarkers revealed a significant difference between *S. uberis*-positive and control group, by clustering into two groups and showing different directions in their expression or methylation changes (Fig. 8B). *SMPD3*, *SLC40A1* and 10 DMCs had higher expression or methylation levels in healthy control group, while all the dMHBs, 4 DE genes and 5 DMCs were highly methylated or expressed in *S. uberis*-positive group (Fig. 8A). The loading weight of each discriminant biomarker indicates its discrimination importance (Fig. 8C). Among the 6 genetic biomarkers (DE genes), *SLC40A1* is of highest importance (loading weight = 0.97) and its expression is about 5 times higher in the healthy control group (log_2_FC = − 2.28, FDR = 9.82 × 10^−7^). For epigenetic biomarkers, all 5 dMHBs show higher methylation levels in the *S. uberis*-positive group. A dMHB in Chr5 (chr5:32984279:32984330) was the most important dMHB biomarker (loading weight = − 0.80), followed by dMHBs on Chr2 (chr2:27911955:27911972, loading weight = − 0.39) and Chr24 (chr24:7353387:7353442, loading weight = − 0.32). A dMHB on Chr5 (chr5:32984279:32984330) overlapped with repeat element ALTR2_BT, and the expression level of *PCED1B* gene in its downstream (+ 35,429 bp) was 3.32 times higher in the healthy control group, but not significantly different (log_2_FC = − 1.73, FDR = 0.39). dHMBs chr2:27911955:27911972 and chr24:7353387:7353442 are located in the intron regions of *STK39* and *CD226*, respectively. Two-thirds of all selected DMCs showed higher methylation levels in healthy control group, with DMC chrX_123509578 being the most important (loading weight = 0.44). The second and third most important DMC biomarkers were located on Chr26 (chr26_7684695) and Chr14 (chr14_67946960), and within *PRKG1* and *PTDSS1* genes, respectively and having higher methylation levels in *S. uberis*-positive group. Interestingly, two hypomethylated DMC biomarkers (chr1_116798477 and chr1_116798702) were located in the same gene, *MED12L*, which was also up-regulated (log_2_FC = 2.03, FDR = 0.027) in *S. uberis*-positive group. In addition, these two DMCs were also located in the downstream region of *GPR87*.

**Table 1:** Candidate biomarkers distinguishing *S. uberis* positive cows from control (healthy) cows: six DE genes and five dMHBs

DE genes^**a**^	Chr^**b**^	log_**2**_FC^**c**^	FDR^**d**^	Importance^**e**^	dMHBs^**f**^	MHL-diff^**g**^	FDR	Importance	Overlapping elements
LOC104972351	Chr5	5.76	3.45E-08	− 0.02	chr2:27911955:27911972	0.21	1.08E-02	−0.39	*STK39*
LOC107131803	Scaffold	3.09	2.78E-07	−0.15	chr8:112623075:112623120	0.22	1.37E-02	−0.25	Intergenic
*SLC40A1*	Chr2	−2.28	9.82E-07	0.97	chr5:32984279:32984330	0.26	1.37E-02	−0.80	ALTR2C_BT^h^
*SMPD3*	Chr18	−2.75	1.15E-06	0.16	chr19:7781358:7781427	0.22	2.02E-02	−0.20	*COIL*
*TCF7L2*	Chr26	2.49	2.74E-06	−0.04	chr24:7353387:7353442	0.35	2.09E-02	−0.32	*CD226*
LOC112447173	Chr1	6.84	6.34E-06	−0.11					

^a^DE gene: differentially expressed genes between *S. uberis*-positive and negative groups; ^b^chr: chromosome, ^c^log_2_FC: log_2_ transformed fold change in gene expression between the two groups; ^d^FDR: False discovery rate correction according to Benjaminin and Hockberg; ^e^Importance: loading weights calculated by DIABLO method indicates the importance of corresponding selected DE gene or dMHB. The negative value indicates higher DE gene expression or dMHB methylation levels in *S. uberis*-positive group, while the positive value represents lower DE gene expression or dMHB methylation levels in *S. uberis*-positive group; ^f^dMHB: differential methylation haplotype block; ^g^MHL-diff: the difference of MHL (methylation haplotype load) between two groups; ^h^repeat element

**Table 2:** Candidate biomarkers distinguishing *S. uberis* positive cows from control (healthy) cows: fifteen DMCs

DMCs^**a**^	Degree of methylation^**b**^	meth.diff^**c**^	*q* value^**d**^	Importance^**e**^	Gene symbol	log_**2**_FC^**f**^	FDR^**g**^	Other overlapping elements^**h**^
chr1_116798477	hypo	−41.94	3.06E-04	0.29	*MED12L*	2.03	2.72E-02	downstream of *GPR87*
chr1_116798702	hypo	−47.50	1.60E-05	0.01	*MED12L*	2.03	2.72E-02	
chr1_81755303	hypo	−22.22	3.61E-02	0.09	*MAP3K13*	2.00	2.49E-02	
chr2_36910011	hypo	−52.63	5.56E-06	0.11	*BAZ2B*	2.40	1.21E-05	BovB^h^
chr5_113635313	hypo	−28.00	4.20E-02	0.21	*ARFGAP3*	1.32	3.11E-02	
chr5_9029509	hypo	−47.37	2.20E-04	0.29	*SYT1*	−4.62	9.72E-03	
chr7_26444558	hyper	65.00	1.55E-06	−0.10	*PRRC1*	1.44	2.68E-02	
chr14_67946960	hyper	35.90	1.03E-02	−0.39	*PTDSS1*	−1.50	4.02E-03	Bov-tA1^h^
chr15_28521852	hypo	−50.00	1.08E-04	0.21	*FXYD6*	1.89	8.01E-03	
chr19_22014105	hyper	27.27	4.42E-02	−0.16	*NXN*	2.34	5.96E-03	
chr19_33218275	hypo	−27.50	2.08E-02	0.28	*TRPV2*	−2.46	1.39E-07	L2a^h^
chr20_56637267	hypo	−30.00	4.92E-02	0.13	*RETREG1*	2.38	1.84E-06	
chr21_61174672	hyper	54.55	1.32E-05	−0.28	*ATG2B*	1.93	8.14E-04	L1ME3C^h^
chr26_7684695	hyper	43.59	1.48E-03	−0.40	*PRKG1*	3.77	3.41E-04	
chrX_123509578	hypo	−26.32	3.49E-02	0.44	*SH3KBP1*	−1.90	1.84E-04	

^a^DMC: differentially methylated cytosines in the context of CpG; ^b^hypo and hyper represents hypomethylated and hypermethylated, respectively, in *S. uberis*-positive group; ^c^meth-diff: difference in the methylation level of DMC between groups. The negative value indicates lower expression and positive value indicates higher expression in *S. uberis*-positive group; ^d^*q* value: adjusted *p* value for the false discovery rate; ^e^Importance: loading weight calculated by DIABLO method indicates the importance of corresponding selected DE gene or dMHB. The negative value indicates higher DE gene expression or dMHB methylation levels in *S. uberis*-positive group, while the positive value represents lower DE gene expression or dMHB methylation levels in *S. uberis*-positive group; ^f^log_2_FC: log_2_ transformed fold change in gene expression between the two groups; ^g^FDR: False discovery rate correction according to Benjaminin and Hockberg; ^h^indicates repeat elements

## Discussion

Subclinical mastitis caused by different pathogens including *S. uberis* has no visible symptoms making detection harder, and causing significant economic losses. The milk somatic cells indirectly capture the host response to an insult and constitute an effective sample for investing the genetic and epigenetic changes associated with *S. uberis* subclinical mastitis. Moreover, compared with mammary gland tissue or blood, milk somatic cells are easier to collect without causing extra harm or discomfort to milking cows, thus respecting animal welfare and also easier and cheaper to apply in a larger population. However, milk somatic cells are composed of multiple cell types, each of which may contribute differently to the genetic or epigenetic alterations between *S. uberis*-positive and healthy groups. Moreover, the small sample size (3 cows per group), although adequate for the types of sequencing technologies used [47], may cause relatively lower statistical power. Therefore, use of single cell sequencing technology and a higher sample size are necessary to validate our findings and the discriminant biomarkers detected in this study. As far as the authors know, this is the first study to profile the whole genome wide DNA methylation patterns of milk somatic cells from cows with naturally occurring *S. uberis* subclinical mastitis.

The genome-wide DNA methylation pattern of milk somatic cells detected in this study is consistent with other mammals, in that DNA methylation mainly exists in the context of CpG [48] and with methylation levels greater than 70%. This study investigated the DNA methylation patterns in all three contexts (CpG, CHG and CHH) and found significant differences between CpG sites and CHG/CHH sites. CHG and CHH sites did not show a downward trend of methylation levels at the promoter region typical for CpG sites, but displayed higher methylation levels in exons, especially first exons. CHG and CHH sites also showed higher methylation levels in CGI, where CpG sites are usually unmethylated. This opposite trend in methylation level distribution of CHG and CHH sites from CpG sites suggests that the mechanisms of CHG and CHH methylation involvement in mastitis may be different from CpG methylation, deserving further exploration. At a genome wide scale, the methylation level of the promoter region showed significant negative correlation with gene expression level, thus supporting a repression role of DNA methylation at the promoter region on transcriptional activities [49–51]. Additionally, this study further found that, the higher the expression level of a gene, the lower its methylation level is at the TSS, and the more intense the decrease in methylation level is at the promoter region (Fig. 6D). This further proved the importance of a low level of methylation at the promoter region for normal gene expression.

The methylation status of milk somatic cells was then compared between *S. uberis*-positive and healthy control group. Firstly, the CpG methylation landscape among chromosomes indicates globally a lower methylation level in *S. uberis*-positive group. Specifically, more than half of the identified DMCs had lower methylation levels in *S. uberis*-positive group when compared to the control group. In support of our data, a lower DNA methylation level was also reported in blood neutrophils of cows with *E. coli*-induced mastitis [22] and mammary epithelial cells challenged with bacterial lipopolysaccharide [52], as well as in cow ileum tissue positive for *Mycobacterium avium* spp. *paratuberculosis* infection [53]. As expected, more DMCs were in the CpG context, further supporting the important regulatory roles of CpG methylation during disease processes. It is worthy of note that about half the identified DMCs were located in repeat elements, represented by SINE, LINE and ERV, which is reasonable because repeats account for about half the bovine genome. DNA methylation performs very important roles in the transcriptional silencing of repeats to restrict their genotoxic potentials and thereby contribute to genome stability [54]. It has been suggested that the changes in DNA methylation status in diseases may enable the activation of repeat elements, especially cancers [55]. Thus, the enrichment of DMCs in repeat elements in this study suggests a possible regulatory mechanism of DNA methylation by affecting the activities of repeat elements in response to *S. uberis* infection. In this study more hypomethylated DMCs were observed in CGI shores, which is opposite to results in humans whereby CGI shores hypermethylation has been widely identified in cancer and tumor development [56] and also reported to regulate the expression of key genes in human breast cancer [57, 58].

In this study, the DNA methylation changes during *S. uberis* subclinical mastitis were not only identified at single cytosine sites but also at cytosine regions to avoid the possible technical noise resulting from measuring the methylation levels of single cytosines. Firstly, 316 DMRs were found by scanning the whole genome with fixed-width windows, leading to the identification of important regions with greater methylation differences. However, this method does not consider the interaction between adjacent methylation sites, prompting us to explore additional methods. The DNA methylation changes in response to environmental stressors, especially disease pathogens, are mediated by changes in implicated enzyme activities (DNMT1, DNMT3A/B and TET proteins) [59]. The change in enzyme activities is locally coordinated, leading to similar methylation status of adjacent sites, which contributes to form methylation haplotypes [30]. Therefore, we used the method of MHB (methylation haplotype block) to investigate the co-methylation status of adjacent CpG sites and to identify biologically relevant linked regions of DNA methylation sites known as MHBs. A total of 451 dMHBs showed significant differences in the co-methylation status of adjacent sites between *S. uberis*-positive and control groups, revealing possibly more direct epigenetic changes in response to *S. uberis* infection. For example, a dMHB on Chr5 (chr5:115877805–115,877,881) overlapped with SINE2–1 and harbored three CpG sites that were highly correlated with each other (*r*^2^ > 0.7). The three CpG sites were all unmethylated in the healthy control group but presented methylation levels of about 70% in *S. uberis*-positive group. Another dMHB on Chr11 (chr11:18837854–18,837,864) was very short, being only 10 bp length and harbored 4 fully correlated DMCs (*r*^2^ = 1). Meanwhile, dMHB chr11:18837854–18837864 was unmethylated in *S. uberis*-positive group, but showed mean methylation level of ~ 91.33% in the control group. It is worthy of note that dMHB chr11:18837854–18837864 located in the intron of *CRIM1* has been reported to be involved in the regulation of mammary gland morphology [60] and milk protein concentration [61]. Besides, a *CRIM1* variant has been associated with neutropenia during pediatric acute lymphoblastic leukemia [62], suggesting its possible effects on neutrophil activity, which is important for mastitis pathogenesis. This further highlights the potential of dMHB chr11:18837854–18837864 as a candidate biomarker of *S. uberis* subclinical mastitis. As shown by these two examples, MHBs represents the co-methylation status of a number of adjacent CpG sites, which could help to reduce the possible technical bias that is the main limitation of quantifying single DMC sites. Although DMR profiling is an improvement over single DMC quantification, it also has the limitation of choosing the length of windows and sliding step during identification. These limitations are overcome with the technique of MHB which relies on the relationship between adjacent DMCs and the bias of specifying sequence length is removed. In addition, MHBs are much shorter than DMR making it easier to profile in a larger population. Therefore, MHBs are relatively more reliable to be used as candidate biomarkers than DMC or DMR.

To further investigate the regulatory roles and mechanisms of DNA methylation changes in response to *S. uberis* mastitis, the DNA methylation and gene expression data were integrated. Based on MethGET results, a total of 1623 MetGDE genes were identified with significant changes (MethGET *P*-value < 0.001) in their gene expression levels and/or DNA methylation levels at promoter regions, gene bodies, introns or exons. The DNA methylation alterations of the MetGDE genes possibly regulated the expression changes of corresponding genes, prompting further analyses to understand their potential roles. For instance, the MetGDE gene *TTC9* had 19% higher promoter methylation level and about 3.5 times lower expression level in *S. uberis*-positive group, suggesting its expression was more likely repressed by its increased promoter methylation. Interestingly, a bunch of MetGDE genes are well-known immune-related genes, such as 10 genes of the interleukin family (*IL6*, *IL9*, *IL12B*, *IL17A*, *IL17F*, *IL17RB*, *IL1RN*, *IL27RA*, *IL36B* and *IL36G*) and 24 genes of the solute carrier family (e.g. *SLC2A6*, *SLC24A1* and *SLC25A21,* etc.). Interleukin genes play important roles in inflammation and the immune system, and some of them, such as *IL6* has been found to regulate the immune response to bovine mastitis and also identified as a candidate biomarker of subclinical mastitis [63, 64]. It is worth mentioning that MetGDE gene *SLC25A21* harbored 46 DMCs in its gene body and 6 DMCs at its promoter region, and 80% of them were hypo-methylated. *SLC25A21* has been reported as differentially expressed in mammary gland tissue following infection by *E. coli* and *S. aureus* [65]. The hypomethylation and up-regulated expression of *SLC25A21* in *S. uberis*-positive group in this study suggest that its altered expression during *S. uberis* mastitis may be regulated by DNA methylation changes.

Functional enrichment of MetGDE genes in GO terms and KEGG pathways with immune and disease related functions, further supports the potential regulatory roles of DNA methylation of these genes and their involvement in the immune response during *S. uberis* mastitis. In particular, a cluster of annotations related to cytokine process and activities were enriched with potentially up-regulated activities (positive Z-score), including Cytokine-cytokine receptor interaction, Chemokine activity, Chemokine-mediated signaling pathways, Cellular response to interleukin-1 and Chemokine signaling pathway. Chemokines, such as interleukins, interferons and chemokines, play key roles in the regulation of intensity and duration of inflammatory, and immune responses to mastitis infection by regulating the activities of immune-related cells [15, 16]. The significant enrichment of these cytokine-related pathways strongly suggest that DNA methylation changes are potentially involved in the regulation of cytokine activities by mediating the expression of related genes, and the host immune response to *S. uberis* infection. Interestingly, a cluster of highly related MetGDE genes with chemokine activities, including *CXCL2*, *CXCL8*, *CXCL12*, *CCL3*, *CCL4*, *CCL5* and *CCL20*, were enriched in multiple pathways mentioned above. These genes are highly related to each other and involved in similar immune processes [66, 67], and their altered activities have been associated with the host response to mastitis. For example, *CXCL2* and *CXCL8* have been identified as the most informative genes and as candidate biomarkers of bovine mastitis, suggesting their possible effects on cow’s ability to resist mastitis [65, 68–70]. Besides, *CCL3*, *CCL4*, *CCL5* and *CCL20* have also been reported as differentially expressed in response to mastitis by other investigators [71–73]. The *CCL5* gene has been found to be up-regulated in bovine mammary epithelial cells stimulated by *E. coli*, but down-regulated in mammary glands with *S. aureus*-induced mastitis [66, 74]. Moreover, *CCL5* has also been reported to be closely associated with the pathogenesis of chronic mammary inflammation and mastitis [67, 75]. These reports highlight the important association of these genes with mastitis; however, the underlying regulatory mechanisms are not well understood. The DNA methylation changes in these genes during *S. uberis* infection observed in this study and during mastitis caused by other pathogens [17, 20, 21, 76], suggest DNA methylation changes as one of the underlying regulatory mechanisms. An in vitro challenge of immortalized bovine mammary epithelial cells with peptidoglycan and lipoteichoic acid induced global hypomethylation by regulating DNMT activity and causing inflammation [19]. In agreement, the DNA methylation of *S. uberis*-positive group was globally lower than of the control group in this study. Furthermore, the expressions of *CXCL2*, *CXCL8*, *CXCL12*, *CCL3*, *CCL4*, *CCL5* and *CCL20* were up-regulated in *S. uberis*-positive group, while hypomethylation was detected in the promoter and gene body regions of most of them (except *CXCL2*). This indicates that DNA methylation changes may be one possible mechanism that modulated the differential expression of these key genes leading to the activation of the activities of chemokines and heightened host immune response to *S. uberis* infection. Another cluster of significantly enriched GO term and KEGG pathways related to diseases and immune response pathways was represented by NF-κB signaling pathway and Antigen processing and presentation pathway. The NF-κB pathway plays roles as the upstream signal that controls the transcription of the inflammatory factors mentioned above [77, 78]. Besides, the activity of the antigen processing and presentation pathway tended to be increased (positive Z-score), suggesting that the enriched MetGDE genes (e.g. *CD40LG*, *LOC524810*, *BOLA-DQA2*, *LOC100300716* and *MGC126945)* may be involved in the regulation of the adaptive immune response by affecting antigen processing. For example, the interaction between *CD40LG* and *CD40* is very important for immune responses dependent on T cells, and they have been found to participate in bovine inflammatory responses [79]. *LOC524810* (IgM) and *LOC100300716* (immunoglobulin heavy variable 4–38-2) are immunoglobulins performing critical functions of recognizing and binding antigens, such as *S. uberis*. Genetic variations in *BOLA-DQA2,* a Bovine Leukocyte Antigen (BOLA) class II gene, have been associated with resistance to dairy cow mastitis [80]. MGC126945, an uncharacterized protein, was found as highly variable in bovine respiratory disease [81].

DNA methylation mediates genomic adaption to environmental influences without changing the underlying DNA sequence and consequently contributing to phenotypic expression, and throwing more light on explaining the “black box” between the phenotype and the genotype [82]. Including epigenetic biomarkers to current livestock breeding programs may improve the prediction accuracy of genetic breeding values and thereby increased genetic gain [82–84]. Furthermore, it could contribute to complement genomic information and provide a better understanding of the factors that shape livestock phenotypes and directional application in breed improvement and management practices [85]. A large body of evidence from human studies reveals DNA methylation changes as promising biomarkers for disease diagnosis or risk assessment [86, 87]. Therefore, this study utilized the discriminant analysis of DIABLO that allows integration of DNA methylation and gene expression data [39], to identify candidate biomarkers (DE genes, DMCs and dMHBs) of *S. uberis* mastitis. The candidate biomarkers delineated clearly the two groups, suggesting their ability to discriminate *S. uberis*-positive cows from the control group. The candidate biomarkers consisted of genetic biomarkers (6 DE genes), and epigenetic biomarkers (15 DMCs and 5 dMHBs). The epigenetic biomarkers were highly correlated with genetic biomarkers (cor > |0.80|), suggesting that DNA methylation changes (epigenetic biomarkers) are more likely involved in the expression changes of the genetic biomarkers. Looking at the epigenetic candidates, all 5 dMHBs showed higher methylation levels in *S. uberis*-positive group. The most important dMHBs (chr5:32984279:32984330 and chr24:7353387:7353442) and four DMC candidates were overlapped with repeat elements, suggesting that epigenetic candidate biomarkers may be involved in the regulation of the host response to *S. uberis* infection by mediating transposition activities. For instance, dMHB chr5:32984279:32984330 overlapped with repeat element ALTR2C_BT, belonging to ERV1 family. Endogenous retroviruses (ERV) are ubiquitous in mammalian genomes, and some have been found to play roles as enhancers for immune-related genes in both human and mice [88]. In addition, dMHB chr24:7353387:7353442 overlapped with transposon L1MA9, a member of L1 family, as well as *CD226* gene which functions by mediating cytotoxicity and is associated with immunologic diseases [89, 90]. The function of repeat elements in bovine diseases, including mastitis is ill-defined. Our data suggest involvement in epigenetic processes, which requires further exploration. Among the candidate DE genes, *SLC40A1* has the greatest loading weight and its correlation with epigenetic biomarkers was extremely high (cor > |0.99|). *SLC40A1* was significantly down-regulated in *S. uberis*-positive group and harbored three hypermethylated DMCs in its gene body, suggesting that gene body methylation may be involved in the regulation of its expression. On the contrary, the *SLC40A1* promoter was identified as hypomethylated with associated up-regulated mRNA expression in blood neutrophils, and consequently considered a candidate biomarker for improving resistance to bovine mastitis induced by *E. coli*, [22]. These contrasting effects of *SLC40A1* DNA methylation on its gene expression may be caused by the differences in tissue or the pathogenic bacteria between the two studies. Differential immune responses and related genetic or microRNA regulation has been reported during infections by *Staphylococcus aureus* and *E. coli* [69, 74, 91]. This suggests that the DNA methylation status and expression of *SLC40A1* may change in different directions during mastitis caused by *S. uberis* compared with *E. coli*.

## Conclusion

The whole genome DNA methylation landscape and transcriptomes of bovine milk somatic cells indicated global negative correlations between CpG methylation level and gene expression at not only the promoter regions but also first exon and first intron regions. Methylation haplotype blocks that considers the co-methylation status of adjacent CpG sites were also identified through the whole genome to enrich the DNA methylation patterns. The DNA methylation alterations explored at different layers between cows with *S. uberis* subclinical mastitis and healthy control indicated that: (1) globaly, the DNA methylation level of *S. uberis*-positive group was lower than healthy control; (2) the DNA methylation changes identified included 174,342 DMCs (in the context of CpG, CHG and CHH), 316 DMRs, and 415 dMHBs; and (3) the DNA methylation changes of 1623 MetGDE genes were related to their gene expression changes, as well as significantly enriched in biological processes and pathways related to the immune response and disease processes, especially cytokine activities, indicative of regulatory roles in the host immune response to *S. uberis* infection. Finally, a total of 26 candidate biomarkers (6 DE genes, 15 DMCs and 5 dMHBs) were identified by discriminant and correlation analyses to clearly distinguish *S. uberis*-infected cows from healthy controls. These candidate genetic and epigenetic biomarkers may serve as reference materials for designing mastitis control, management and breeding strategies.

## Supplementary Information

**Additional file 1: Fig. S1.** DNA methylation level trends. (**A**) Bean plots showing the methylation level distribution of methylation sites in the context of CpG, CHG and CHH at genome wide scale. (**B**) The methylation level trends around gene features per sample. a = promoter (2 kb upstream of transcription start site); b = first exon; c = first intron; d = inner exons; e = inner introns; f = last exon; g = downstream (2 kb downstream of transcription termination site. (**C**) The methylation level trends around CpG islands (CGI) per sample. CGI shores are the 2 kb regions flanking the CGI. CGI shelf left is the 2 kb region upstream of CGI shore left while CGI shelf right is the 2 kb region downstream of CGI shore right.

**Additional file 2: Fig. S2.** Methylation status of differentially methylated sites. (**A-B**) Volcano plots showing DMCs in the context of CHG (**A)** and CHH (**B**). (**C**) Bar plots showing distribution of differences in methylation levels of DMCs.

**Additional file 3: Fig. S3.** Annotation of DMCs. (**A-C**) The annotation of DMCs in the context of CpG (**A**), CHG (**B**) and CHH (**C**). (**D**) The distribution of DMC CpG islands (CGIs) and related regions. (**E-F**) The number of DMCs grouped by the differences in methylation levels in gene features (**E**) and regions in CGI context (**F**).

**Additional file 4: Fig. S4.** DNA methylation landscape. (**A**) The number of DMCs grouped by the differences in methylation levels per chromosome. (**B-D**) Manhattan plots showing the global distribution of DMCs in the context of CpG (**B**), CHG (**C**) and CHH (**D**). (**E**) DNA methylation landscape of chromosome 2.

**Additional file 5: Fig. S5.** Association between DNA methylation and gene expression levels. (**A-B**) Scatter plots showing correlation between gene expression level and promoter methylation level (**A**) and first exon (**B**). (**C**) Box plot showing promoter methylation level of genes grouped by expression levels. (**D-F**) Scatterplots and fitting curves of DNA methylation and relative gene expression of gene body methylation (**D**), exon methylation (**E**) and intron methylation (**F**). (**G-I**) MetGDE genes selected based on the methylation changes of gene body (**G**), exons (**H**) and introns (**I**). (**J**) Venn diagram showing the number of unique and shared promoter-, gene body-, exon- and intron- MetGDE genes.

**Additional file 6: Table S1.** Genes and their primer sequences used for real-time qPCR validation of RNA-sequencing results.

**Additional file 7: Table S2.** Read mapping statistics of whole-genome bisulfite sequencing data and RNA sequencing data of six cows.

**Additional file 8: Table S3.** The list of differentially methylated cytosines (DMCs) in the context of CpG, CHG and CHH.

**Additional file 9: Table S4.** The number of DMCs (CpG, CHG and CHH) in different genetic regions.

**Additional file 10: Table S5.** The list of DMRs and annotation.

**Additional file 11: Table S6.** The list of genes harboring DMCs or overlapping with DMRs.

**Additional file 12: Table S7.** Functional enrichment of genes harboring at least 15 DMCs.

**Additional file 13: Table S8.** The list of differentially methylated haplotype blocks (dMHBs) and functional enrichment for hyper dMHBs.

**Additional file 14: Table S9.** The list of differentially expressed (DE) genes and their functional enrichment.

**Additional file 15: Table S10.** The genome-wide correlation between DNA methylation and gene expression and list of MetGDE genes.

**Additional file 16: Table S11.** The results of functional enrichment differentially methylated and expressed genes (MetGDE genes).

**Additional file 17: Table S12.** The cluster of functional annotations enriched by differential methylated and expressed genes (MetGDE genes).

**Additional file 18: Table S13.** The correlation between discriminant candidate biomarkers.

## Data Availability

The datasets generated and/or analyzed during the current study are available in the NCBI Sequence Read Archive (SRA) under the BioProject: PRJNA835098 (whole genome DNA methylome data) and BioProject: PRJNA835097 (RNA-Seq data).

## Abbreviations

BP: Biological process
CC: Cellular component
CGI: CpG island
CHG: Cytosine methylations in CHG sequence, where H correspond to A, T or C (A = adenine; T = thymine; C = cytosine)
CHH: Cytosine methylations in CHH sequence, where H correspond to A, T or C
Chr: Chromosome
CpG: 5′-C-phosphate-G-3′ (C = cytosine; G = guanine)
CTG: Control group
DE genes: Differentially expressed genes
DMCs: Differentially methylated cytosines
dMHBs: Differential methylation haplotype blocks
DMRs: Differentially methylated regions
*E. coli*: *Escherichia coli*
ERV: Endogenous retrovirus
FDR: False discovery rate
GCCA: Generalised canonical correlation analysis
GMM: Gaussian mixture model
GO: Gene Ontology
HSCC: High milk somatic cell count
KEGG: Kyoto Encyclopedia of Genes and Genomes
LINE: Long interspersed nuclear element
log_2_FC: log_2_ fold change
LSCC: Low milk somatic cell count
MetGDE genes: Genes with significant changes in their methylation levels and/or gene expression changes
MF: Molecular function
MHBs: Methylation haplotype blocks
MHL: Methylated haplotype load
non-LTRs: Non-long terminal repeats
PBS: Phosphate buffered saline
PLS: Projection to latent structure model
RIN: RNA integrity number
*S. uberis*: *Streptococcus uberis*
SCC: Milk somatic cell count
SINE: Short interspersed nuclear element
SUG: *S. uberis*-positive group
*TLR4*: Toll-like receptor 4
TSS: Transcription start site
