# Supplementary figures and images for: Methylome and transcriptome data integration reveals potential roles of DNA methylation and candidate biomarkers of cow Streptococcus uberis subclinical mastitis
Source: J Anim Sci Biotechnol. 2022 Nov 7;13:136. doi: 10.1186/s40104-022-00779-z (PMC9639328; doi:10.1186/s40104-022-00779-z)

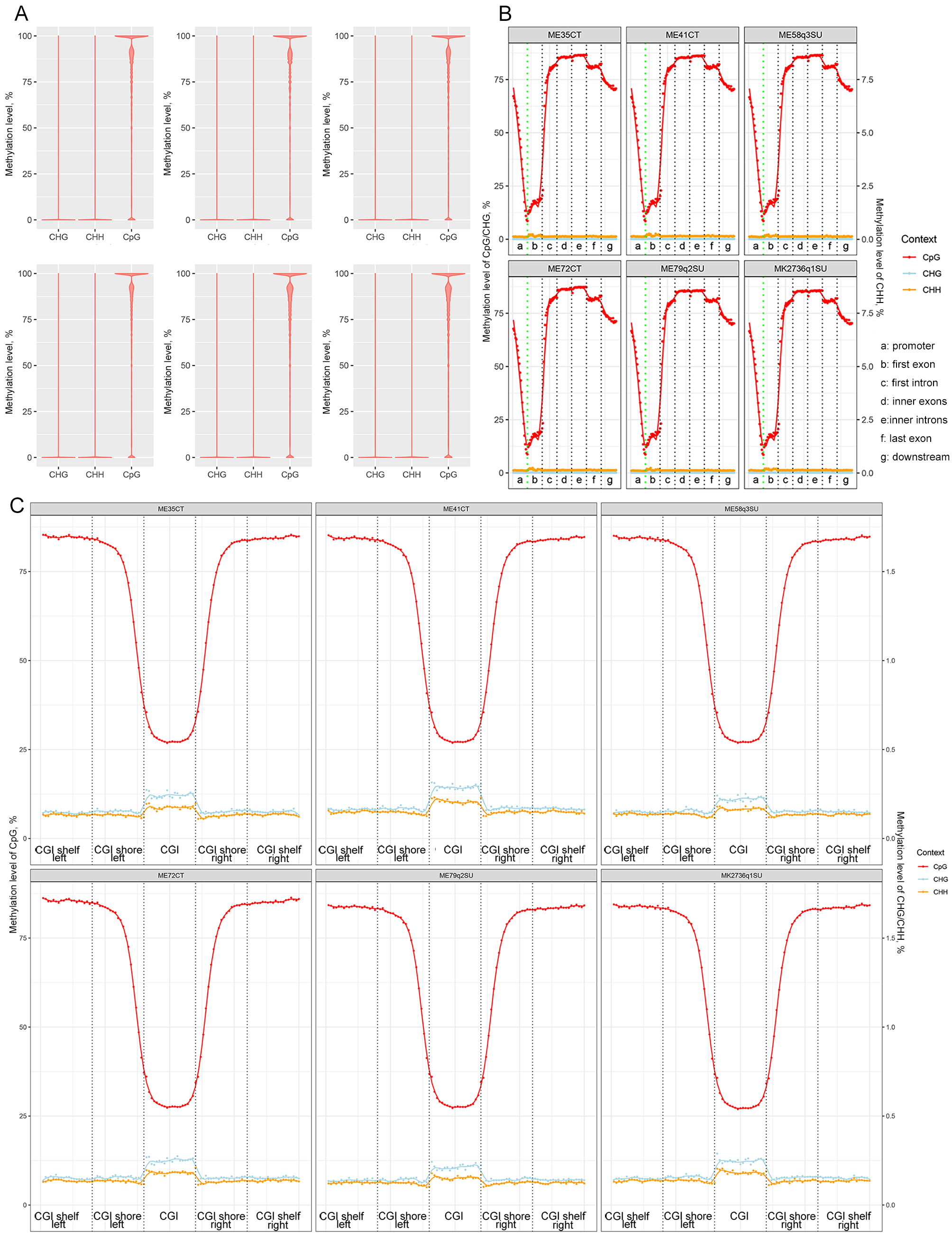

Supplement: Supplementary file 1 — Additional file 1: Fig. S1. DNA methylation level trends. (A) Bean plots showing the methylation level distribution of methylation sites in the context of CpG, CHG and CHH at genome wide scale. (B) The methylation level trends around gene features per sample. a = promoter (2 kb upstream of transcription start site); b = first exon; c = first intron; d = inner exons; e = inner introns; f = last exon; g = downstream (2 kb downstream of transcription termination site. (C) The methylation level trends around CpG islands (CGI) per sample. CGI shores are the 2 kb regions flanking the CGI. CGI shelf left is the 2 kb region upstream of CGI shore left while CGI shelf right is the 2 kb region downstream of CGI shore right. [file 40104_2022_779_MOESM1_ESM.tif]

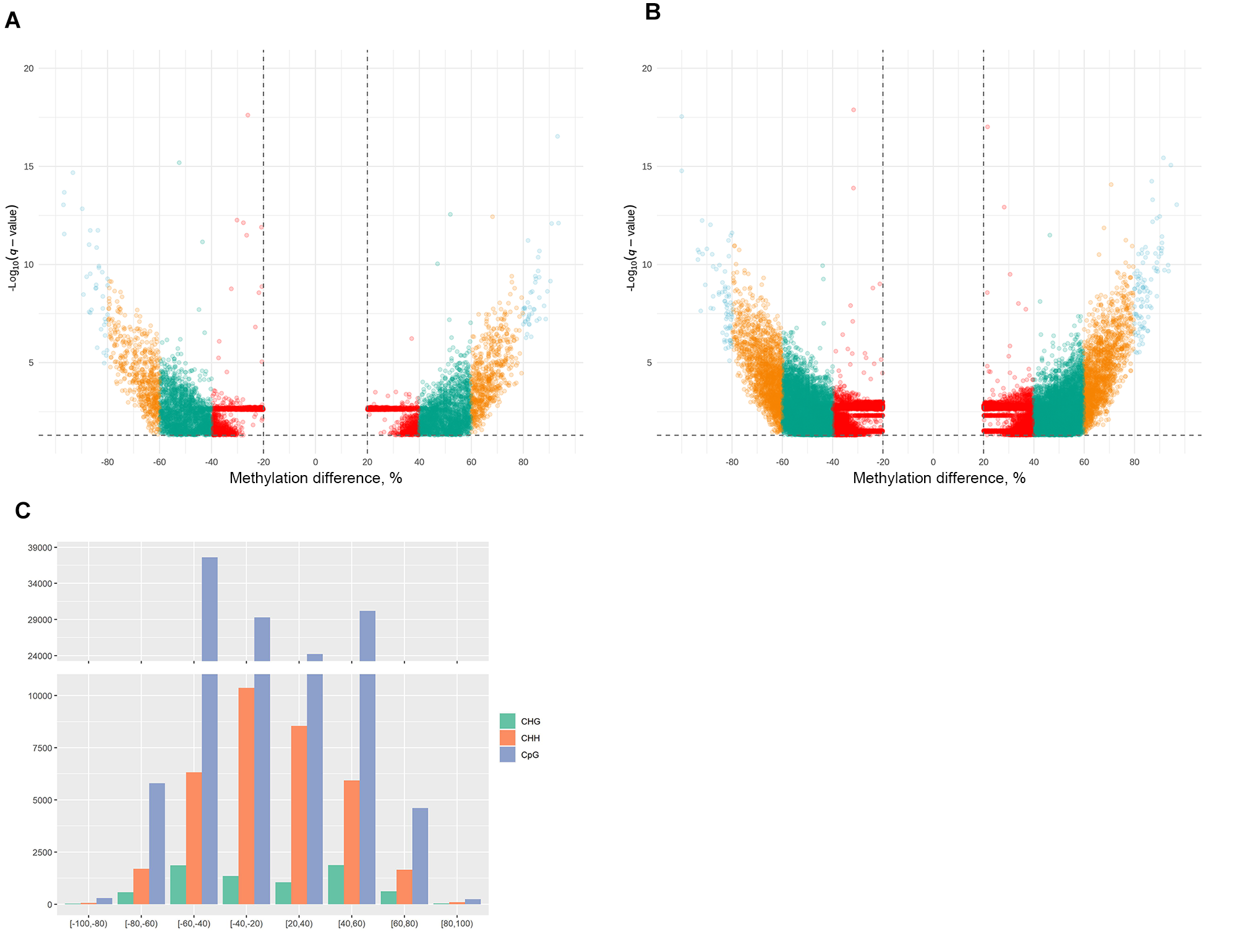

Supplement: Supplementary file 2 — Additional file 2: Fig. S2. Methylation status of differentially methylated sites. (A-B) Volcano plots showing DMCs in the context of CHG (A) and CHH (B). (C) Bar plots showing distribution of differences in methylation levels of DMCs. [file 40104_2022_779_MOESM2_ESM.tif]

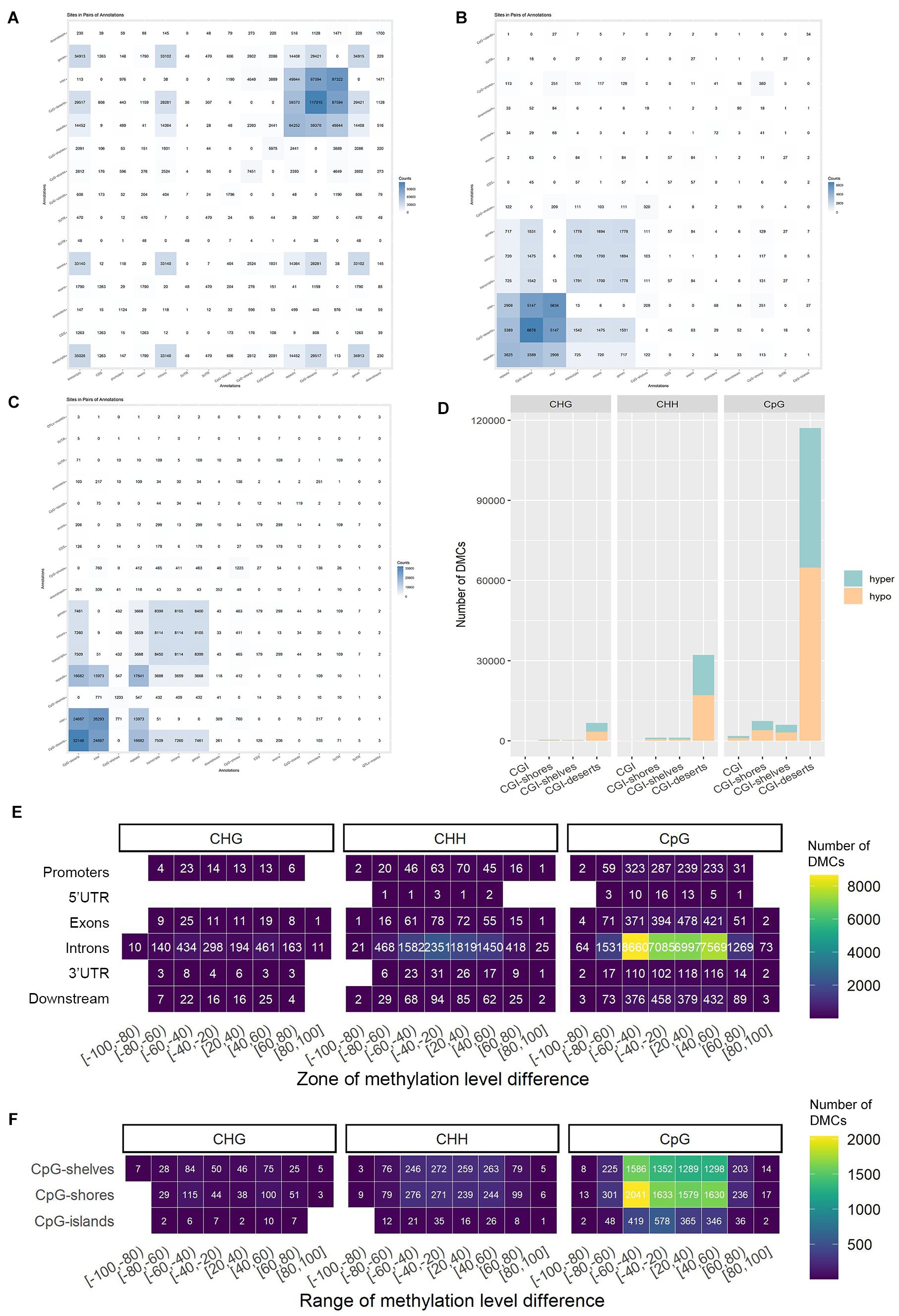

Supplement: Supplementary file 3 — Additional file 3: Fig. S3. Annotation of DMCs. (A-C) The annotation of DMCs in the context of CpG (A), CHG (B) and CHH (C). (D) The distribution of DMC CpG islands (CGIs) and related regions. (E-F) The number of DMCs grouped by the differences in methylation levels in gene features (E) and regions in CGI context (F). [file 40104_2022_779_MOESM3_ESM.tif]

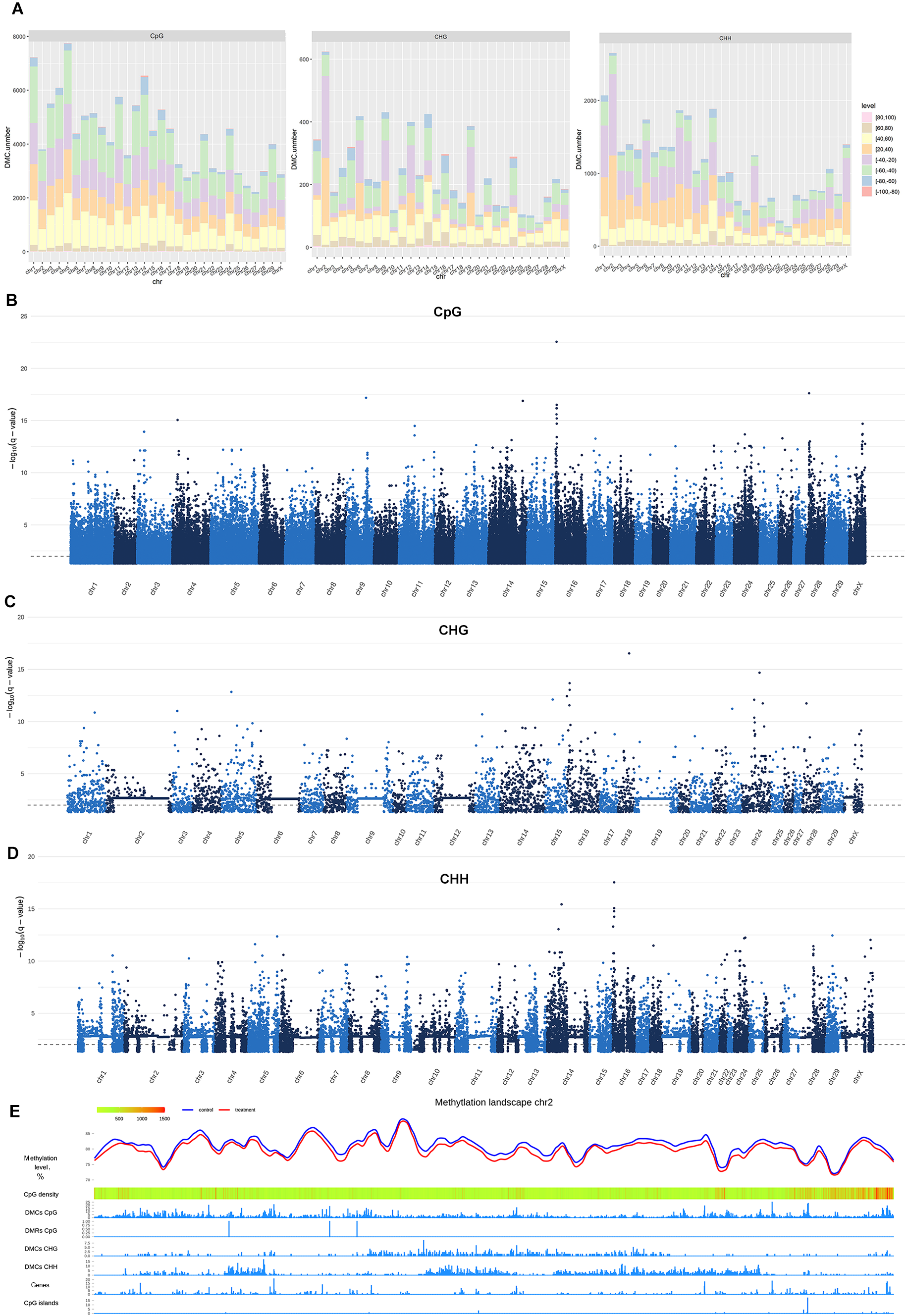

Supplement: Supplementary file 4 — Additional file 4: Fig. S4. DNA methylation landscape. (A) The number of DMCs grouped by the differences in methylation levels per chromosome. (B-D) Manhattan plots showing the global distribution of DMCs in the context of CpG (B), CHG (C) and CHH (D). (E) DNA methylation landscape of chromosome 2. [file 40104_2022_779_MOESM4_ESM.tif]

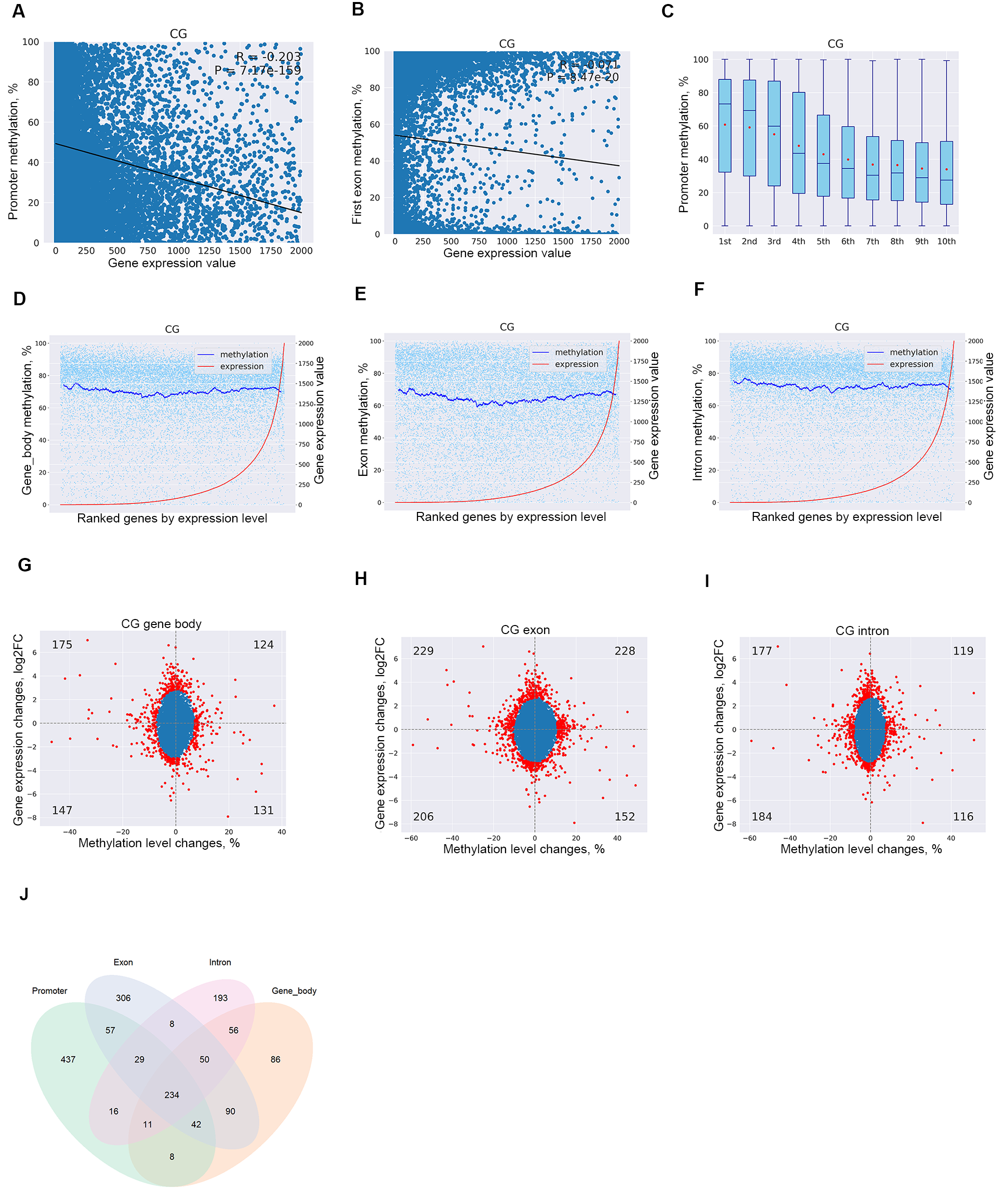

Supplement: Supplementary file 5 — Additional file 5: Fig. S5. Association between DNA methylation and gene expression levels. (A-B) Scatter plots showing correlation between gene expression level and promoter methylation level (A) and first exon (B). (C) Box plot showing promoter methylation level of genes grouped by expression levels. (D-F) Scatterplots and fitting curves of DNA methylation and relative gene expression of gene body methylation (D), exon methylation (E) and intron methylation (F). (G-I) MetGDE genes selected based on the methylation changes of gene body (G), exons (H) and introns (I). (J) Venn diagram showing the number of unique and shared promoter-, gene body-, exon- and intron- MetGDE genes. [file 40104_2022_779_MOESM5_ESM.tif]
